# Toxicity of Some Natural Products in the Treatment of Rheumatoid Arthritis

**DOI:** 10.3390/life16081319

**Published:** 2026-08-12

**Authors:** Keyla Nunes Farias Gomes, Raíssa Maria dos Santos Galvão, Natalia Lidmar von Ranke, Carlos Rangel Rodrigues, Caroline de Souza Ferreira Pereira, Julianne Soares Pereira, Matheus Amorim Rosa e Silva, Brenda Bairral Queiroz Ornellas, Jonathas Albertino de Souza Oliveira Carneiro, Geovana Espindola Jardim, André Lopes Fuly, José Augusto Albuquerque dos Santos, Robson Xavier Faria

**Affiliations:** 1 Postgraduate Program in Plant Biotechnology and Bioprocesses, Center of Health Sciences, Federal University of Rio de Janeiro, Carlos Chagas Filho Avenue 373—University City, Rio de Janeiro 21941-902, RJ, Brazil; kfarias1618@gmail.com; 2 Laboratory of Environmental Health Assessment and Promotion, Oswaldo Cruz Institute, Av. Brasil 4365, CEP, Rio de Janeiro 21040-900, RJ, Brazil; carolinesfp@id.uff.br (C.d.S.F.P.); universodigital.tj@gmail.com (J.A.d.S.O.C.); geovanaej@id.uff.br (G.E.J.); santosjaa@gmail.com (J.A.A.d.S.); 3 Postgraduate Program in Science and Biotechnology, Institute of Biology, Fluminense Federal University, Valonguinho Campus, Niterói 24220-900, RJ, Brazil; raissa.sgalvao@gmail.com (R.M.d.S.G.); juliannesope@gmail.com (J.S.P.); amorimmatheus123@gmail.com (M.A.R.e.S.); brendabairral@gmail.com (B.B.Q.O.); andrefuly@id.uff.br (A.L.F.); 4 Molecular Modeling and 3D-QSAR Laboratory, Faculty of Pharmacy, Federal University of Rio de Janeiro, Rio de Janeiro 21941-901, RJ, Brazil; nataliavonranke@hotmail.com (N.L.v.R.); rangelfarmacia@gmail.com (C.R.R.); 5 Laboratory of Animal Venoms and Toxins and Evaluation of Inhibitors, Department of Cellular and Molecular Biology, Institute of Biology, Fluminense Federal University, Gragoatá Campus, Niterói 24210-201, RJ, Brazil

**Keywords:** rheumatoid arthritis, natural products, treatment, toxicity, in silico analysis

## Abstract

Rheumatoid arthritis (RA) is a chronic autoimmune disease that affects mainly peripheral joints because of inflammation of the synovial membrane. Current treatments, such as nonsteroidal anti-inflammatory drugs (NSAIDs) and glucocorticoids, although effective, are associated with high costs and several adverse effects. In this context, natural products have emerged as promising alternatives because of their potential therapeutic effects and lower toxicity. The objective of this review was to identify and evaluate natural substances with potential applications in RA treatment on the basis of studies published between 2015 and 2020. A literature search was conducted in SciELO, PubMed, and Google Scholar using the keywords “rheumatoid arthritis”, “treatment”, “toxicity”, and “natural products”. Additionally, we applied in silico methods to predict pharmacokinetic and toxicological parameters using ADMET Predictor^®^ (Simulation Plus) and compared the results with those of commercial drugs such as diclofenac, ibuprofen, and naproxen. Target fishing (reverse docking) was also performed to identify possible molecular targets related to RA. Seven natural compounds were identified, mostly evaluated through in vivo studies. Among them, paeoniflorin, quercetin, resveratrol, and celastrol are in clinical phases and present potential as RA treatments. In silico analysis highlighted curcumin, tetramethylpyrazine, and resveratrol as the most promising candidates, with ADMET profiles comparable or superior to those of current NSAIDs. In conclusion, natural products represent viable alternatives for RA therapy. However, further studies are essential to better understand their safety, pharmacokinetics, and drug interactions to ensure their clinical applicability.

## 1. Introduction

RA is an autoimmune disease that affects approximately 1% of the global population. As a chronic, progressive disease affecting peripheral joints, RA causes changes in quality of life because of joint damage [1,2].

The main feature of this disease is inflammation of the inner lining of the joints, which is called symmetrical synovitis. In the initial phase of the disease, an acute inflammatory response occurs, which soon becomes a mechanism of chronic joint destruction [2,3].

This inflammatory disease can present manifestations in other organs, such as the lungs, vessels and even skin, and in the joints. RA is a major generator of comorbidities, affecting smaller joints more frequently, such as those between the hands, feet, and fingers. In a short period of time, the joints are destroyed, causing chronic pain and subsequent loss of function. Patients affected by RA usually have a higher incidence of other diseases simultaneously [3,4]. As described by Semerano et al. (2016) [5], this disease has a high degree of limitation, and treatment should be started quickly, especially in the first 12 months after the onset of symptoms, especially in the first 12 weeks, which is considered the therapeutic window. During this period, treatment may be most effective at delaying and preventing the onset of comorbidities.

To date, there are 4 known treatments for RA: nonsteroidal anti-inflammatory drugs (NSAIDs), glucocorticoids (GCs), and disease-modifying antirheumatic drugs (DMARDs), in addition to biological agents, such as inhibitors of tumor necrosis factor, T-cell costimulatory blocking agents, B-cell reducing agents, interleukin-6 inhibitors, and interleukin-1 receptor antagonists [6,7,8].

The purpose of this treatment should be to relieve pain, decrease the degree of inflammation, slow the process of cartilage destruction, improve well-being, prevent joint formation and reduce the need for surgical intervention.

However, many drugs used for the treatment of RA, in addition to having a high cost for the patient, can cause a wide range of adverse effects, such as renal, hepatic, and cardiovascular toxicity. Additionally, diabetes, hypertension, hepatitis, aplastic anemia, pneumonia, tuberculosis, and candidiasis can occur. Thus, new effective and safer therapeutic agents for the treatment of this autoimmune disease are necessary [7,9].

One of the problems with conventional therapy used in rheumatoid arthritis (RA) patients is the long-term use of disease-modifying antirheumatic drugs (DMARDs) and the high cost of approximately USD 26,000 per year. Owing to the prolonged use of these drugs, patients with RA are at greater risk of adverse side effects, especially when they are associated with intensive pharmacological management [10]. In addition, the economic burden of RA treatment involves multiple components, including costs related to pharmacological therapies, medical consultations, diagnostic monitoring, and long-term disease management. Patients with RA may also incur additional expenses associated with complementary and alternative therapies, such as dietary supplements and other biologically based products used for symptom management [10].

Herbal medicines have been widely used to complement and assist in the treatment of rheumatic diseases, such as arthritis, mainly because of their anti-inflammatory effects [11]. However, the lack of scientific evidence concerning the efficacy and toxicity of these medicinal plants in treating RA has led to insecurity, thus reducing their use [12].

Dietary supplements from natural products, defined as substances derived from nonmineral, systemically ingested natural products, are the most frequently used form of complementary and alternative medicine in the United States, with approximately 18% of American adults reporting the use of these products [10].

Natural products derived from plants have become popular in the pharmaceutical industry because of their minimal adverse effects, in addition to their targeted pharmacological activities. These products are widely found in herbs, vegetables, fruits, and seeds that are commonly consumed in the human diet [13].

Natural products have been shown to be effective at treating various inflammatory diseases because of their anti-inflammatory, antioxidant, and apoptotic activities. Additionally, delaying bone resorption has been shown to be effective for the treatment of rheumatoid arthritis [13].

Consequently, understanding the current scenario of drugs from natural products to treat rheumatoid arthritis can aid in the development of therapeutically effective substances with fewer adverse effects over short or long periods.

Thus, we reviewed some works in the literature that used substances from natural products with efficacy for the treatment of rheumatoid arthritis over a period of five years between 2015 and 2020.

## 2. Materials and Methods

**Figure 1:**
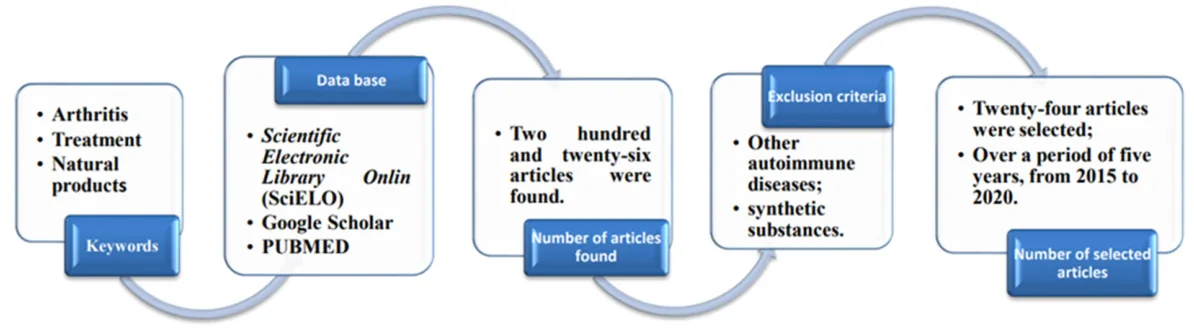
Descriptive organization chart of the article selection process in the database.

A search was performed in the scientific databases Scientific Electronic Library Online (SciELO), Google Scholar, and PubMed. The period between 2015 and 2020 was investigated using the keywords “rheumatoid arthritis”, “treatment”, “toxicity”, and “natural products”. A total of 226 articles were identified. Among these, 24 studies met the objective of this review, addressing the effects of natural products in the treatment of rheumatoid arthritis (RA). The inclusion criteria included original and review articles published during the selected period that specifically evaluated natural products for RA treatment, including their therapeutic effects and toxicity profiles. Studies focusing on other autoimmune diseases, synthetic compounds, duplicated records, and articles not available in full text or not directly related to the scope of this review were excluded. Because of the selected studies, we analyzed the effects of natural products in the treatment of rheumatoid arthritis, including toxicity predictions and their main mechanisms of action (Figure 1).

## 3. Computational Analysis of Isolated Substances

### 3.1. In Silico Assay

The physicochemical and toxicological profiles were predicted via ADMET Predictor^®^ (Simulation Plus). We used the commercial anti-inflammatory drugs diclofenac, ibuprofen and naproxen for comparison.

### 3.2. Target Fish

During the first stage, potential molecular targets of rheumatoid arthritis ligands were identified. Virtual screening techniques, in which this applied method is called “target fishing”, which uses methodologies to search for similarity between ligands and the base receptor (reverse docking), were used. These analyses were performed in layers to seek screening and funneling of data directed toward molecular targets that result in a greater probability of interaction with the compounds involved [14].

Therefore, we used a search based on similarity to explore a database with a stipulated quantity of 3000 mammalian molecular targets, along with a reverse docking analysis stage with the 50 most promising molecular targets.

## 4. Results and Discussion

Since antiques were developed, natural products have been used to treat or improve the symptoms of several diseases [12]. Herbal medicines have assisted in the treatment of rheumatic diseases, such as arthritis, especially because of their anti-inflammatory effects [11]. However, scientific data concerning the effects of these herbal medicines in patients with RA are scarce, generating insecurity and reducing their use [12].

Studies on herbal medicines suggest a promising alternative approach for the treatment of inflammatory processes, including those associated with rheumatoid arthritis (RA) [15].

Recent research suggests that genetic and environmental factors influence the development and progression of RA, which is characterized by synovitis, hyperplasia, and destruction of bone and cartilage. The pathogenesis of RA involves an inflammatory response, and several biological agents have been developed to treat this disease. However, the activity of these biological agents is limited by uncertain pathophysiological responses and other side effects [16].

Hepatotoxicity is among the side effects of common and alternative treatments for RA and is a concern of regulatory agencies because of the withdrawal of these drugs from the market. This is related to the scarcity of data on the toxicity of herbal medicines in the literature [17]. In contrast, several herbal medicines have been investigated as potential therapeutic agents to assist in the treatment of RA (Table 1, Table 2 and Table 3).

## 5. Chemical Composition and Toxicity of Natural Production

### 5.1. Crude Extract

#### 5.1.1. *Salix alba*

*Salix alba*, popularly known as white willow, is a small tree native to Europe, Asia, and North Africa that belongs to the family Salicaceae. This species is very common in cold and low-temperature regions of the Northern Hemisphere and Poland, where it grows mostly along riverbanks in moist soils [18].

The main component extracted from *S. alba* is salicin, which is a metabolic precursor of salicylic acid with similar activity to that of aspirin and has antipyretic and analgesic effects. Other phenolic compounds found are salicylic acid, salidroside, saligenin, tremulodine, salicoilsalicin, salicortin and tremulacin, which act synergistically at sites of inflammation [19].

Although adverse effects are common for patients who already use more than one drug, *Salix alba* may have some side effects if it is not used correctly or at the correct dosage. Some authors described the association of *S. alba* with paracetamol-induced nephrotoxicity due to the salicylate present in the plant [12].

A double-blind, randomized, and controlled study evaluated the effects of *Salix alba* L. bark extract for two weeks. Patients with osteoarthritis received 240 mg/salicin/day, which had a moderate analgesic effect compared with that of the placebo. Additionally, the recommended therapeutic dose of *S. alba* is 200 mg to 300 mg daily [12].

This plant is contraindicated for children and teenagers under 18 years of age and patients with asthma, active peptic ulcer disease, severe cases of liver or kidney dysfunction, coagulation disorders, gastric ulcers/duodenal ulcers, glucose-6-phosphate dehydrogenase deficiency, and allergies to salicylates. *Salix alba* should also be avoided during pregnancy and lactation because of the reduction in uterine tone and motility [12].

With respect to the toxicity of the *S. alba* extracts, the mice were administered three different doses (500, 1000, or 2000 mg/kg) daily for seven consecutive days. After the treatment period, neither the treated nor the control groups exhibited signs of toxicity, such as mortality, weight loss, lethargy, or apparent morbidity. Cell viability was assessed using a comet assay in combination with trypan blue staining, and no treatment-related alterations were observed. This assay was further employed to evaluate DNA strand breaks in peripheral blood leukocytes, as well as in heart, liver, testicular, and bone marrow cells from Swiss mice. No significant increase in DNA migration was detected in either the control or treated groups [11]. Additionally, the authors investigated genotoxic effects by analyzing the frequency of micronucleated polychromatic erythrocytes (MNPCEs) in mouse bone marrow cells. The results showed that *S. alba* extract did not significantly alter the MNPCE frequency at any tested dose. In contrast, the positive control (doxorubicin, 30 mg/kg) induced a significant increase in MNPCE levels. The ratio of polychromatic erythrocytes to normochromatic erythrocytes (PCE/NCE) was also evaluated, and no significant changes were observed following treatment with S. alba extract, suggesting the absence of hematopoietic toxicity [20].

In the study by Coelho et al. (2018) [12], the potential clinical effects of willow bark were evaluated in patients with arthritis. Gastrointestinal disorders were the most commonly reported adverse events, followed by peripheral and central nervous system disturbances, as well as skin and appendage alterations. However, no severe adverse effects were reported. Notably, no significant differences in the risk of adverse effects were observed when *S. alba* extract was compared with analgesic or placebo treatments. Previous studies have indicated that willow bark is commonly associated with mild gastrointestinal symptoms, including nausea, upper abdominal pain, dyspepsia, and gastric discomfort. Furthermore, the potential to accumulate heavy metals, along with reports of gastrointestinal upset and rare anaphylactic reactions, should be considered when evaluating its safety profile [21].

#### 5.1.2. *Tripterygium wilford* II

*Tripterygium wilfordii* is among the most studied natural products because of its therapeutic effects on RA [7] and is commonly known as the Thunder of God Vines. This plant species belongs to the Celastraceae family and is geographically distributed in humid areas of the Northern Hemisphere, including China, Japan, South Korea, North Korea, and North America [22].

Approximately 380 metabolites were identified from *Tripterygium wilfordii* extracts, 95% of which are terpenoids [22]. In addition, more than 500 chemical constituents, including sesquiterpenes, diterpenes, triterpenes, alkaloids, flavonoids, lignans, and glycosides, have been isolated and identified from *T. wilfordii*. This extract has several pharmacological activities, mainly anti-inflammatory, anticancer, antiviral and antioxidant activities [23].

*T. wilfordii* may suppress T/B-cell proliferation and the growth of synovial fibroblasts and induce T-apoptosis [9]. Both diterpenoids and the triterpene celastrol exhibit anti-inflammatory activities.

Celastrol may reduce the inflammatory response of synovial fibroblasts by activating calcium signaling and inhibiting cell proliferation through the induction of DNA damage, cessation of the cell cycle, and promotion of apoptosis. In addition, both celastrol and tryptopy can inhibit the migration of synovial fibroblasts and invasion by suppressing the expression of metalloproteinase 9 from the NF-κB-mediated matrix (MMP-9) and blocking the activation of the JNK-MAPK pathway [22,24].

The use of *T. wilfordii* can cause fetal malformation. For this reason, the use of this plant should not be recommended for women who are in a fertile period and who want to become pregnant [12].

In a randomized clinical trial, the objective was to compare the efficacy and safety of *T. wilfordii* and methotrexate in the treatment of active RA. Two hundred seven patients with active RA were treated with 12.5 mg of methotrexate once a week, 20 mg of *T. wilfordii* three times a day, or both in combination. The results indicated that monotherapy with *T. wilfordii* and monotherapy with methotrexate presented similar effects. However, combination therapy has shown greater efficacy than monotherapy with methotrexate [22].

Using molecular docking and network pharmacology approaches, Mao and Xie (2024) [25] investigated the potential mechanisms of *Tripterygium wilfordii*, revealing multiple signaling pathways and molecular targets associated with its therapeutic effects. Among the active compounds, triptolide strongly binds to key inflammatory targets, including TNF, PTGS1, and PTGS2, suggesting its potential as a multitarget inhibitor.

In addition, glycosides extracted from the roots of *T. wilfordii* have shown promising effects in the treatment of rheumatoid arthritis (RA), mainly because of their immunosuppressive and anti-inflammatory properties. Zhang et al. (2021) [22] reported that these compounds ameliorate arthritis in rats with type II collagen-induced arthritis by reducing IL-1β production and anti-CII autoantibody levels and decreasing arthritis scores, paw swelling, and joint damage [26].

Despite these therapeutic benefits, *T. wilfordii* is associated with significant toxicity. Hepatotoxic effects have been reported, including clinical manifestations such as hepatomegaly, jaundice, dark urine, loss of appetite, fatigue, and elevated serum levels of alanine aminotransferase (ALT) and aspartate aminotransferase (AST) [27].

Furthermore, toxicological studies indicate that *T. wilfordii* may impair reproductive function in both males and females, affecting sperm quality, epididymal and testicular structure, and ovarian function. It may also induce hematological toxicity, leading to damage to platelets and leukocytes and negatively affecting the urinary and cardiovascular systems [22].

#### 5.1.3. *Pterodon* spp.

The trees of the genus *Pterodon* spp., known as ‘white sucupira’ or ‘faveiro’, are native to Brazil and belong to the botanical family Fabaceae. Owing to their anti-inflammatory and analgesic activities, the fruits produced by this plant are ingested in small amounts for the treatment of various diseases, including RA. There are little data in the literature, and those found often do not describe adverse reactions or drug interactions [12,28].

Phytochemical studies in the genus *Pterodon* have demonstrated the presence of isoflavones, sesquiterpenes and diterpenes in its fruit oil and other metabolites, such as isoflavones and triterpenes, alkaloids, saponins, glycosides and steroids, sesquiterpenes, isoflavones and saponins, in wood, tree bark and leaves, respectively. The main compounds isolated from Pterodon species are linear and tetracyclic diterpenes with vouacapano skeletons, which partially underlie the pharmacological activities of fruit-derived oils [28].

The part of the plant used was the bark in the form of an extract, and the compound of pharmacological action was beta-caryophyllene. This compound acts in the chain of cyclooxygenases, causing the entire inflammatory chain to be blocked, thus achieving pain relief [12].

With respect to the toxicity of *Pterodon pubescens* oil, Pacheco and collaborators reported low toxicity to peripheral blood mononuclear cells (IC_50_ of 2 μg/mL) after acute oral administration in male DBA1/J mice. The oil doses administered (2, 4 and 8 g PpSO/kg) were much higher than those used in traditional medicine [29]. Dosage and mode of commercial use of this herbal medicine: dry extract—400 mg twice daily; fluid extract—0.5 to 2 mL per day; powder—500 mg twice daily; tincture—2 to 10 mL per day, 20 drops three times a day; infusion or decoction—6 seeds every 1.5 L of water [29].

#### 5.1.4. Pomegranate, Grape Extract and Propolis

Pomegranates, grape extracts and propolis have been widely studied because of their possible use in many pathologies, including those of inflammatory origin. According to a qualitative study, the main constituents, such as phenolic acids, flavonoids, and stilbenes, are promising for RA treatment because of the strong anti-inflammatory activity reported in in vitro and in vivo studies [30].

Propolis is obtained by collecting the exudates of different plant species. This compound has a complex mixture of resin, waxes, balm, and pollen and a small proportion of secondary metabolites. Propolis is known for its antiviral, antimicrobial, immunomodulatory, and anti-inflammatory activities, which inhibit IL-17 production and Th17 cell differentiation [30].

Pomegranates of the species *Punica granatum* L., Punicaceae family, and grape extracts of the species *Vitis vinifera* L., family Vitaceae, have been studied for the treatment of RA because a variety of chemical compounds, such as alagic acids (elagitanins), phenylpropanoids, and flavanoids, derived from cinnamic acid, present immunomodulatory and anti-inflammatory effects [30].

Female mice aged 8–12 weeks received an emulsion containing a mixture of pomegranate, grape extract, and propolis. The mice were treated with this mixture in an injectable form (50 μL per mouse). Disease severity was evaluated weekly and individually, and the symptoms and degree of severity were observed [30].

According to the authors, treatment with a mixture of these natural products improved swelling and decreased the incidence and symptoms in mice. Early treatment with pomegranate, propolis, or the grape extract mixture prevented the increase in the levels of IL-17 and IL-1β in the mice. These plant products have eased the severity of symptoms and are plausible options for the treatment of RA.

Heo et al. (2022) [31] studied the relationship between rheumatoid arthritis fibroblast-like synoviocytes (RA-FLSs) and grape seed proanthocyanidin extract. The authors demonstrated that compared with the ROS inhibitor N-acetylcysteine, this extract significantly reduced intracellular reactive oxygen species (ROS) levels, suggesting that propolis is a potential therapeutic compound for the treatment of RA.

Propolis has also been associated with cardioprotective effects in patients with RA, contributing to a reduction in cardiovascular risk. Its atheroprotective properties are related to the inhibition of atherosclerotic plaque formation, as well as reductions in liver cholesterol, hepatic triglyceride, and circulating triglyceride levels in experimental models. These effects are likely linked to its beneficial effect on lipid metabolism. In addition, propolis exhibits significant antioxidant activity, reducing oxidative stress levels. It also decreases nitric oxide production, thereby protecting endothelial cells through the modulation of nitric oxide synthase (NOS) activity in blood vessels [32].

#### 5.1.5. *Chenopodium ambrosioides*

The species *Chenopodium ambrosioides*, from the Amaranthaceae family, is popularly known as’matruz. This plant is widely distributed in Brazil and has several pharmacological properties, such as antiprotozoal, anti-inflammatory, and antibacterial properties and a reduction in synovial inflammation [33].

Pereira et al. (2018) [33] analyzed the hydroalcoholic crude extract of *C. ambrosioides* leaves through phytochemical analysis and high-precision liquid chromatography with a diode array detector (HPLC–DAD). They detected the presence of secondary metabolites, such as flavones and flavonol derivatives, phenolic compounds, and quercetin and kaempferol derivatives.

According to Pereira et al. (2018) [33], the secondary metabolites found in *C. ambrosioides* leaves have different functions that could directly contribute to RA treatment. For example, quercetin has different uses (applications), such as in the treatment of angiogenesis, cancer, infections, cartilage deformities and bone.

Arthritis was induced by collagen in DBA/1J mice, and the treatment was given orally (5 mg/kg) 21 days after the induction of arthritis for 6 weeks. The results indicated a good antiarthritic effect of the crude extract of *C. ambrosioides* leaves, with inhibition of the serum concentrations of IL-6 and TNF-α. This effect is related to the inhibition of the production of proinflammatory cytokines, thus protecting the joints against possible bone density loss [33].

#### 5.1.6. *Pterocarpus santalinus*

*Pterocarpus santalinus* from the Fabaceae family is popularly known as red sandalwood. In antiquity, wood pulp has been used to treat boils, skin rashes, infections, inflammation, and headaches. Recently, several pharmacological properties, such as antibacterial and anticancer properties, have been described for treating inflammation, ulcers, headaches, and healing wounds [34].

Dhande et al. (2017) [34] performed a qualitative phytochemistry study of the species *P. santalinus*. They described the presence of anthocyanins, carbohydrates, tannins, phenols, glycosides, triterpenoids, and lignans. According to previous studies, savinin, which is a lignan that inhibits the production of TNF-α and T-cell proliferation, can act as an active ingredient and induce anti-inflammatory effects.

For the experiments, albino rats of both sexes were used. Chronic inflammation was induced with complete Freund’s adjuvant (CFA) in the subplantar tissue of the left paw of each rat. After treatment with a paste or gel, the mice were evaluated on days 0, 12 and 28 [34].

In this study, several parameters, such as the body weights of the animals, paw measurements and pain assessments, were analyzed. The authors observed a reduction in body weight and paw volume in rats treated with *P. santalinus* gel compared with those in the groups that did not receive the plant. Additionally, pain was reduced in all the groups, but this parameter was greater in the gel-treated group [34].

#### 5.1.7. *Schinus terebinthifolius*

*Schinus terebinthifolius* is a species of plant commonly known as ‘aroeirinha’, Brazilian pepper or red-bellied pepper. This species belongs to the Anacardiaceae family. *S. terebinthifolius* is popularly used to treat skin, inflammation, ulcers, gout, tumors, respiratory problems, and arthritis. According to previous studies, the crude and fractional extracts of this species are rich in polyphenols and have antibacterial, antiallergic, and antioxidant properties [35].

Rosas et al. (2015) [35] performed phytochemical analyses (chromatographic adsorption column, thin layer chromatography, partition chromatography, gas chromatography coupled with mass spectrometry, high-efficiency liquid chromatography and crystallization) on hydroalcoholic extracts of the species *S. terebinthifolius.* They detected several main constituents, such as polyphenols, gallic acid, methyl gallate and 1,2,3,4,6–pentagalloylglucose.

The anti-inflammatory effect of the hydroalcoholic extract of *S. terebinthifolius* leaves (ST-70) was investigated in an experimental model of zymosan-induced inflammation. Male Swiss and C57BL/6 mice pretreated orally with ST-70 (3125–200 mg/kg) 1 h before zymosan induction were used. Treatment with ST-70 at a mean effective dose (ED_50_) of 100 mg/kg inhibited 70% of neutrophil accumulation and reduced the joint diameter and neutrophil influx to synovial tissues at 6 and 24 h in zymosan-induced arthritis [35].

ST-70 also inhibited the production of IL-6, IL-1β, keratinocyte-derived chemokine (CXCL1/KC) and tumor necrosis factor (TNF-α) at 6 h and produced CXCL1/KC and IL-1β at 24 h. In addition, daily treatment (20 days) with ST-70 did not induce gastric damage or death in the animals. This effect did not occur in the group treated with a similar dose of diclofenac, which induced gastric damage and killed all the animals on the fifth day of treatment [35].

Hydroalcoholic extracts of the leaves of the species *S. terebinthifolius* have high potential for the treatment of inflammatory diseases, such as RA. This occurs because of the production of cytokines and chemokines as well as the inhibition of neutrophil recruitment [35].

#### 5.1.8. Kan-Lu-Hsiao-Tu-Tan (Klhtt)

In a study conducted by Chiang et al. (2020) [36], the therapeutic effects of the aqueous extract of Kan-Lu-Hsiao-Tu-Tan (KLHTT), a Chinese medicine based on a mixture of herbs, on RA were analyzed. They used a model of collagen-induced arthritis in which male DBA/1J mice aged six to eight weeks were immunized with chicken CII and analyzed for histological arthritis severity, proinflammatory cytokine levels, oxidative marker concentrations, anti-collagen type II antibodies, and splenocyte proliferation [36].

The immunization of mice with CII increased the clinical arthritis score, paw volume and degree of histopathological damage. Treatment with KLHTT (50 and 100 mg/kg) for 42 days decreased the severity of arthritis, erythema and swelling of the paw. Body weight loss in CIA mice was also restored by the KLHTT. Histopathological analysis revealed that mice treated with KLHTT presented well-preserved joint spaces with minimal inflammatory exudate, normal cartilage structure, clear synovial spaces and improved histological arthritis severity scores [36].

Treatment with KLHTT (50 and 100 mg/kg) decreased the IL-1β, IL-6, IL-17 and TNF-α levels in paw homogenates and serum samples from CIA mice. These results indicated that the KLHTT effectively attenuated inflammation in CIA mice. The treatment also reduced oxidative damage and significantly suppressed the production of IgG1 and IgG2 antibodies. In addition, KLHTT significantly inhibited the proliferation of CII-induced splenicocytes [36].

These results indicate that KLHTT has notable anti-inflammatory, antioxidant, and immunomodulatory effects in CIA mice, supporting its potential use as an alternative or supportive treatment for RA.

## 6. Isolated Substance

### 6.1. Cannabidiol

**Figure 2:**
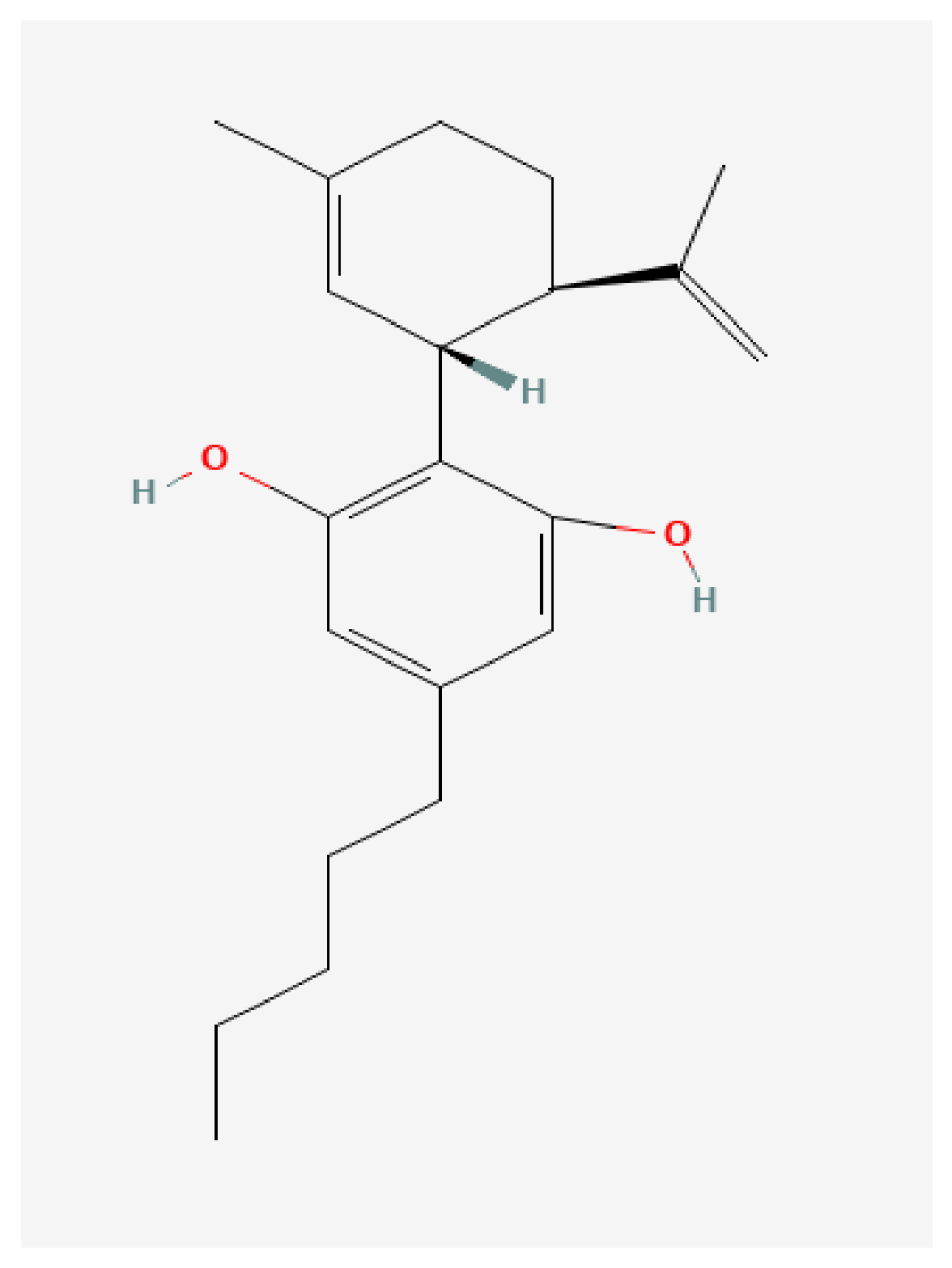
National Center for Biotechnology Information. PubChem Compound Summary for CID 644019, Cannabidiol. https://pubchem.ncbi.nlm.nih.gov/compound/Cannabidiol (accessed on 5 July 2021).

*Cannabis sativa* is a plant species of the Cannabaceae family, commonly called marijuana, and cannabis is found in several habitats and altitudes, possibly originating in the Himalayas [37]. This plant contains more than 500 bioactive compounds, including more than seventy different cannabinoids [38], with cannabidiol (CBD) (Figure 2) being the main nonintoxicating cannabinoid and the most promising phytonabinoid owing to its nonpsychotropic effects, low toxicity and high tolerability [39].

This plant can produce more than 400 compounds (such as terpenoids, flavonoids, and amino acids) that include more than 60 different phytocannabinoids. The biological effects of most of these compounds have not yet been adequately characterized [39].

Cannabis is utilized to treat several diseases, including RA. However, some authors have indicated that this plant may induce drug interactions [12].

Two thousand sixty-nine people completed a questionnaire about the medical use of *Cannabis sativa* L. Almost one quarter of the respondents mentioned the relief of symptoms of RA as a main reason for smoking parts of the plant *Cannabis sativa* L. Additionally, in Australia, in 2005, one study with 128 people reported “great relief” in general (86%) and substantial relief of specific symptoms, such as pain, nausea, and insomnia [39].

The ability of cannabidiol (CBD) (an agonist of the cannabinoid receptor) treatment to treat arthritis induced by collagen was tested in mice. Treatment effectively blocked the progression of the disease. CBD is equally effective when it is administered intraperitoneally or orally, with effects of 5 mg/kg daily or 25 mg/kg daily [39].

CBD is metabolized mainly by the isoenzymes cytochrome P450 (CYP) 2C119 and CYP3A4, which are activated by several antiepileptic drugs (for example, carbamazepine, topiramate, and phenytoin) and inhibited by others (for example, valproate) [40]. Therefore, interactions with other drugs have been investigated.

Cannabidiol has no side effects. However, this consumption is limited to pharmacological extracts, and the plant may have some side effects because CBD affects the central nervous system [12]. For example, in a randomized trial, thirty-four patients received dosages of 5, 10, and 20 mg/kg/d of a pharmaceutical formulation of purified cannabidiol, and seven patients received placebo. The most common symptoms of the pharmaceutical formulation of purified cannabidiol were abnormal behavior, somnolence, vomiting, pyrexia, ataxia, sedation, and decreased appetite. Six patients who consumed the pharmaceutical formulation of purified valproate developed high levels of transaminase; liver damage was not induced by drugs, and all patients recovered [40]. CBD has been used orally at dosages between 40 and 1280 mg/day [38].

In contrast to the psychoactive constituent tetrahydrocanabidiol (THC), CBD did not have a direct effect on cannabinoid receptors 1 and 2 (CB1 and CB2) but modulated the effect of agonists, suggesting an allosteric function [41]. Thus, it reduces binding to THC and, without altering behavioral and cognitive functions, has an immunomodulatory effect, increasing the expression of IL-10, which in turn reduces the expression of proinflammatory cytokines such as TNF-α, IL-1β and IL-6 [42,43,44].

Orrin et al. (2018) [40] described a pharmacokinetic study of oral and transdermal CBD in healthy dogs, which received a dosage of 75 mg every 12 h or 150 mg every 12 h. They reported that the bioavailability of CBD is low when CBD is administered orally in dogs or humans, probably because of the strong effect of the first-pass effect on the liver. The half-lives reported in this study were shorter than those reported in a previous crossover study, which evaluated plasmatic data in 6 dogs after intravenous administration of 45 or 90 mg and then 180 mg orally.

More recent studies, such as those by Valentino and Volkow and Schouten et al. (2024) [43], have demonstrated that CBD has therapeutic effects that, in addition to being immunomodulatory and anti-inflammatory, are also neuroprotective by reducing oxidative stress. There is a scarcity of randomized clinical trials, especially multicenter and long-term studies, compromising the robustness of the available evidence and hindering accurate assessments of the safety, efficacy, and standardization of the therapeutic use of CBD [43,44,45].

### 6.2. Curcumin

*Curcuma longa*, popularly known as turmeric, belongs to the botanical family Ziangiberaceae and has been used for centuries in the treatment of chronic inflammatory diseases, including RA [46,47].

**Figure 3:**
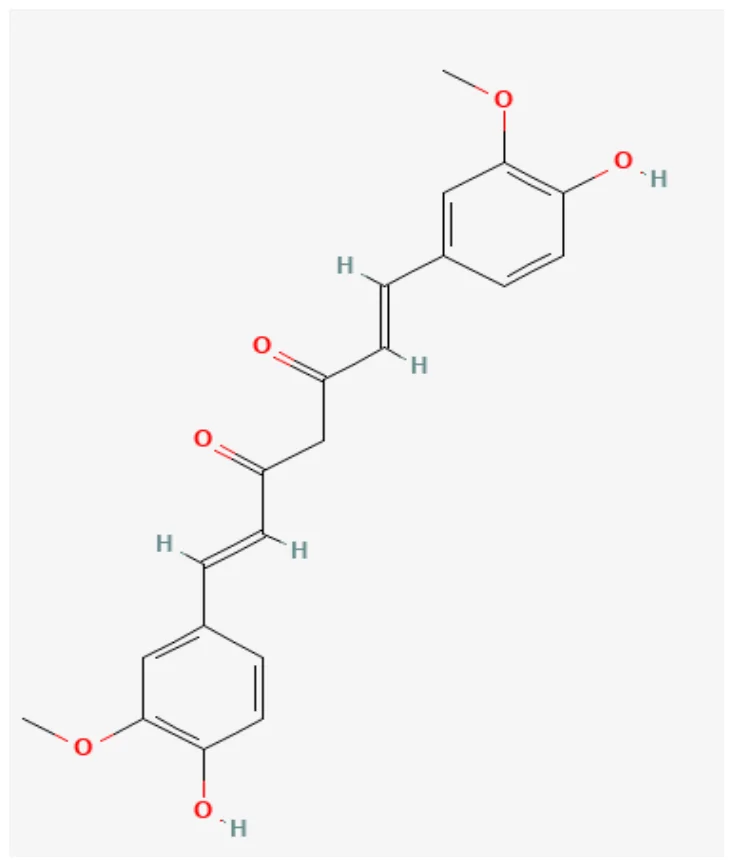
National Center for Biotechnology Information. PubChem Compound Summary for CID 969516, Curcumin. https://pubchem.ncbi.nlm.nih.gov/compound/Curcumin (accessed on 5 July 2021). Curcumin, 1,7-bis(4-hydroxy-3-methoxyphenyl)-1,6-heptadien-3,5-dione, represents 75% of the total amount of *Curcuma longa* L. *extract*, which also contains desmethoxycurcumin structures (curcumin II, 20%) and (III) bisdemethoxycurcumin (curcumin III, 5%) [47].

Curcumin is the main bioactive component of this plant (Figure 3) and appears to promote arthritis by decreasing the number of proinflammatory Th1 and Th17 cells and increasing the number of regulatory T cells. This regulation can suppress collagen-induced arthritis and could be considered an attractive therapeutic strategy for T-cell-mediated autoimmune rheumatic diseases (ARDs), such as RA [47].

*Curcuma longa* can be used for the treatment of RA in the form of gels and lotions, which facilitates the adaptation of patients who are polymedicated. However, patients who use drugs of some pharmacological classes, such as anticoagulants and nonsteroidal anti-inflammatory drugs (NSAIDs), should not use them, as *Curcuma longa* L. may interact with drugs, potentiating and increasing the risk of bleeding [12].

Liu et al. (2026) [48] conducted a systematic review and meta-analysis to evaluate the effects of curcumin and turmeric extract on rheumatoid arthritis (RA) and systemic lupus erythematosus. A reduction in inflammation was observed, suggesting a potential adjuvant therapy; however, the number of trials included was small (n = 6), limiting its effects [48].

Barbosa et al. (2025) [49] reported that the use of turmeric (*Curcuma longa*) alone or in combination with prednisone reduced inflammatory and oxidative effects in female rats with RA, improving the tibiofemoral joint [49].

Although curcumin is described as beneficial for human health, it has low absorption, meaning that a small amount reaches the bloodstream, thus reducing its effectiveness. Through a systematic review, Laurindo and collaborators evaluated several nanoformulations with curcumin that improve bioavailability and thus can be used as an adjuvant medication not only for rheumatoid arthritis but also for Parkinson’s disease, multiple sclerosis, epilepsy, and COVID-19. These nanoformulations can be nanosuspensions, nanoparticles, nanoemulsions, solid lipid particles, nanocapsules, nanospheres, or liposomes [50].

Curcumin is an herbal medicine that is considered safe, as confirmed by several human clinical trials in which this substance did not demonstrate dose-limited toxicity, up to a dosage of 10 g/day. However, there are certain limitations regarding the use of curcumin as a drug. Curcumin has very low bioavailability, and its slow cellular absorption and rapid metabolism restrict its effects. Thus, this compound requires alternatives such as repeated oral doses aimed at a significant concentration inside the cells for physiological effects; consequently, this alternative makes therapy with curcumin difficult [47].

### 6.3. Paeoniflorin (PAE)

**Figure 4:**
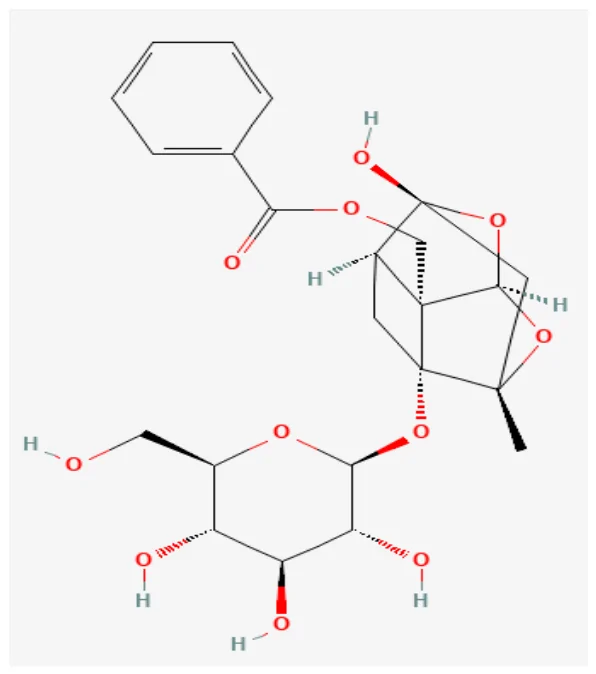
National Center for Biotechnology Information. PubChem Compound Summary for CID 442534, Paeoniflorin. https://pubchem.ncbi.nlm.nih.gov/compound/Paeoniflorin (accessed on 26 June 2021).

Paeoniflorin (PAE) (Figure 4) is a monoterpene glycoside. This compound is purified and extracted from *Paeonia lactiflora*, a Chinese herb often used to treat immune disorders [51,52].

According to Xin et al. (2019) [51], this bioactive compound increased the pain threshold and decreased arthritis symptoms in rats, decreased secondary hind paw swelling and arthritis scores, and reduced the levels of inflammatory cytokines, namely, paeoniflorin. This substance has proven to be a potential compound for RA treatment.

Paeoniflorin has been used for many years in Chinese medicine, mainly because of its anti-inflammatory effects, which have been widely studied. The clinical study included 1784 patients aged between 24 and 72 years. Approximately 20% of patients received paeoniflorin, 20% received antirheumatic drugs, and the rest received other therapies. According to the results of this study, paeoniflorin appears to be a safer option for replacing antirheumatic drugs, which, in the long term, present toxicity. Paeoniflorin has several adverse effects but with a lower frequency and severity than drugs commonly used to treat RA [53].

Jia and He (2015) [54] used paeoniflorin to assess its protective effects in a model of RA. In the experiments, five groups of mice were used to analyze pain thresholds and arthritic symptoms. The doses administered to the mice were 5, 10 and 20 mg/kg over a three-week period. At the end of the experiment, paeoniflorin improved disease in a mouse model of RA through antioxidant and anti-inflammatory effects and changes in COX-2 expression.

Cheng et al. (2016) [55] pharmacologically evaluated monoterpene glycosides derived from the roots of the species *Paeonia lactiflora*. In this study, they used mice and humans to investigate the pharmacokinetics and disposition of these glycosides after intravenous injection of XueBiJing herbs. In the present study, healthy volunteers (men and women) aged between 19 and 31 years were used, and paeoniflorin was responsible for 85% of the monoterpenes detected in the XueBiJing herbal injection. The main route of paeoniflorin elimination occurs via renal excretion in mice and humans. In human studies, paeoniflorin has an elimination half-life between 1.2 and 1.3 h. According to these results, paeoniflorin is a promising compound of great pharmaceutical relevance.

Li et al. (2016) [56] conducted a clinical study to assess the pharmacokinetic parameters, safety, and tolerability of paeoniflorin after intravenous infusions in patients. This study was conducted with healthy Chinese volunteers (males and females) aged 18–45 years. Patients were divided into two groups: the single-dose phase and the multiple-dose phase. In this pharmacokinetic study, paeoniflorin did not significantly differ between male and female patients. The dose used for this purpose was between 3 and 9 g, which was used for seven consecutive days. Approximately 48% of the paeoniflorin was excreted by the kidneys. According to the authors, some pharmacokinetic and safety profiles will be better analyzed in the next phase, where studies II and III of this substance will be performed.

In an in vitro study, Yang et al. (2024) [57] reported that paeniflorine, through modulation of the hsa_circ_009012/miR-1286/TLR4/NLRP3 axis, inhibited inflammation in synovial fibroblasts in rheumatoid arthritis. These synovial fibroblasts reside in the synovial membrane, providing structural support, producing synovial fluid, and maintaining joint homeostasis. In rheumatoid arthritis, these synovial fibroblasts become proliferative, invasive, and hyperactivated; therefore, the inhibition of the inflammatory response by paenoflurane in patients with RA is promising. However, the need for current clinical articles published between 2023 and 2026 is a limitation, as they do not yet demonstrate real clinical efficacy.

### 6.4. Quercetin

**Figure 5:**
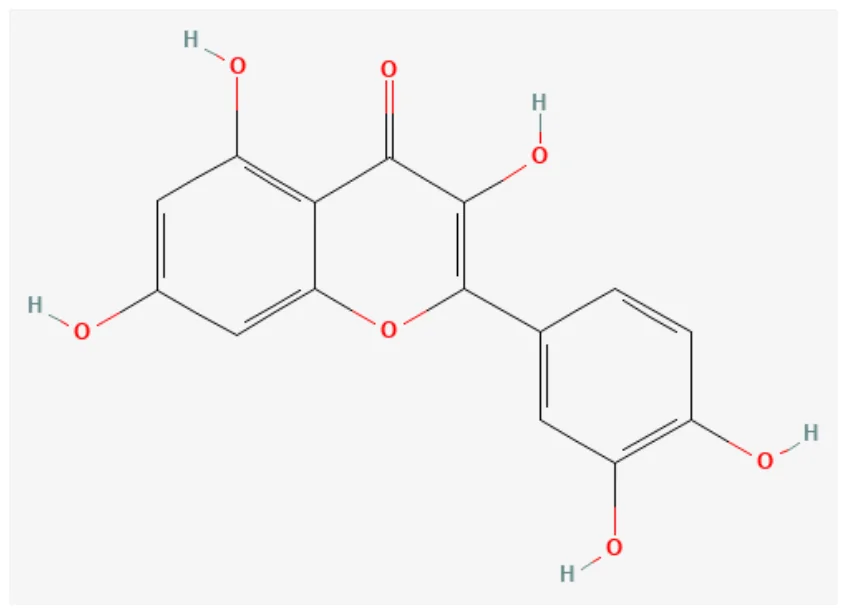
National Center for Biotechnology Information. PubChem Compound Summary for CID 5280343, Quercetin. https://pubchem.ncbi.nlm.nih.gov/compound/Quercetin (accessed on 5 July 2021).

Quercetin (Figure 5) is classified as a flavonol and is one of six subcategories of flavonoid compounds. This compound is the main polyphenolic flavonoid found in many vegetables, fruits and beverages, such as red wine and teas. This substance has a yellow color, is completely soluble in lipids and alcohol, is insoluble in cold water, and is moderately soluble in hot water [58,59].

In 2008, the first study on the pharmacokinetics of quercetin in humans was published, where Moon and colleagues evaluated the pharmacokinetics of quercetin aglycone and its conjugated metabolites. In this clinical study, the subjects received 500 mg quercetin 3 times daily with a meal, and the mean maximum plasma concentration of total quercetin for the 10 subjects was 463 ng/mL at 3.5 h (arithmetic mean). The urinary recovery of quercetin aglycone ranged from 0.05% to 3.6% of the ingested dose, whereas that of quercetin-conjugated metabolites ranged from 0.08% to 2.6%. The plasma concentration versus time curves showed re-entry peaks, suggesting the occurrence of enterohepatic recirculation or discontinuous absorption in humans [59].

In a phase I clinical trial, quercetin was administered by intravenous infusion at increasing doses, initially at 3-week intervals. Fifty-one patients, all of whom were diagnosed with cancer and were no longer amenable to conventional therapies, were included in the study. The initial dosage of 60 mg/m^2^ was chosen on the basis of a previous study with healthy patients and was subsequently increased to 2000 mg/m^2^. The toxic effects observed in some patients were vomiting; cardiovascular effects such as hypertension, chest pain and heart failure; and renal toxicity with increased serum creatine levels. No patient achieved a conventional radiological response according to the WHO criteria. However, one patient with metastatic hepatocellular carcinoma who was treated at a dosage of 60 mg/m^2^ had a sustained decrease in serum α-fetoprotein and phosphatase alkaline levels [60].

Owing to its food-derived origin, the use of quercetin is associated with low toxicity, and its consumption without medical supervision often parallels that of conventional medicines. However, clinically relevant pharmacokinetic interactions of quercetin with drugs have already been demonstrated, both at dietary and supplemental intake levels, owing to quercetin’s ability to modulate drug absorption and metabolism. Nguyen and colleagues investigated the effects of acute and short-term high-dose quercetin ingestion on CYP3A-mediated metabolism using the drug midazolam as its substrate. Coadministration of a single dose of quercetin did not significantly alter the pharmacokinetics of midazolam or its 1′-hydroxymetabolite. However, after the ingestion of quercetin for 1 week, the authors described a trend toward reduced exposure to midazolam. Thus, the authors concluded that coadministration of quercetin in a single dose with midazolam did not cause adverse toxic events. However, repeated ingestion of quercetin may reduce systemic exposure to orally administered drugs, increasing their metabolism, which is catalyzed by CYP3A [61].

Several in vitro studies have demonstrated that quercetin prevents the development of lipopolysaccharide (LPS)-mediated tumor necrosis factor-α (TNF-α) in macrophages and the development of IL-8-induced LPS in A549 lung cells. Furthermore, quercetin can inhibit LPS-induced TNF-α and interleukin (IL)-1α mRNA expression, which results in reduced apoptotic neuronal cell death caused by microglial activation and suppresses the production of inflammatory enzymes (e.g., lipoxygenase (LOX) and cyclooxygenase (COX) [58].

Quercetin was evaluated for its ability to treat RA in a mouse model of adjuvant-induced arthritis. For this purpose, 28 8-week-old C57BL/6 mice were divided into 4 groups: healthy, untreated, quercetin-treated, and dexamethasone-treated patients (1 mg/kg daily). Symptoms of arthritis were observed 3 days after induction, and quercetin was administered intraperitoneally (30 mg/kg) daily from the third day post-induction until 30 days later. Compared with those in the PBS-treated group, the ankle diameters and arthritic scores in the quercetin- and dexamethasone-treated groups were significantly lower. The levels of IFN-γ, TNF, and IL-6 were lower in the quercetin-treated group than in the control group, and neutrophil infiltration and activation were significantly reduced in vivo and in vitro [58].

In a 2024 study, Zhang and colleagues evaluated the immune and inflammatory effects of quercetin on MH74 cells, a human synovial fibroblast line. Quercetin inhibited the activity of IL-6, thus preventing the proliferation of synovial fibroblasts, which exacerbated the inflammatory response and tissue and joint damage. This cascade of events occurs through the activation of the JAK1/STAT3/HIF-1α pathway, which plays a role in the pathogenesis of rheumatoid arthritis (RA). By inhibiting this signaling pathway, quercetin reduces synoviocyte invasion and decreases the secretion of proinflammatory cytokines, thus regulating autoimmune disorders [62].

A 2017 clinical study evaluated the effects of quercetin supplementation on inflammation, severity, and clinical symptoms in women with RA. In this study, 50 women with RA were divided into quercetin (500 mg/day) or placebo groups for 8 weeks, and the plasma levels of high-sensitivity tumor necrosis factor-α (hs-TNFα), erythrocyte sedimentation rate, and clinical symptoms, including morning stiffness, morning pain, tenderness and swollen joints, were recorded. As a result, daily quercetin supplementation for 8 weeks significantly reduced morning stiffness and pain after activity, the plasma level of hs-TNFα was significantly lower in the quercetin group than in the placebo group, and no significant differences in tenderness or swollen joints were observed between the groups. Supplementation affected the erythrocyte sedimentation rate (ESR) but not significantly (*p* > 0.05) [63].

A meta-analysis was conducted in 2023 by Liu and colleagues, and the main results revealed the significant inhibitory effects of quercetin on RA. A reduction in reactive oxygen species; a decrease in cytokines such as C-reactive protein (CRP), TNF-α, IL-1β, IL-6, and IL-17; a reduction in edema; histopathological improvement of the joints; and modulation of inflammatory pathways such as NF-κB were observed. However, the evidence is still limited to preclinical studies, and clinical trials are needed for therapeutic validation [64].

Recently, Zhu et al. (2026) [65] reviewed recent advances in nanomedicine applied to quercetin and its applications in the treatment of osteoarthritis (OA) and rheumatoid arthritis (RA), highlighting strategies to improve its therapeutic efficacy. Although quercetin has protective, anti-inflammatory, and antioxidant potential, it is a substance that has low solubility and bioavailability, rapid metabolism, and, consequently, accelerated elimination from the body. With respect to nanomedicine, which offers advantages in strategies to overcome such limitations, this study addresses various types of quercetin nanoformulations (such as polymeric and lipid nanoparticles) that exhibit low toxicity, reduce inflammatory infiltration, lower TNF-α and IL-6 levels, and can be combined with other drugs, such as methotrexate, allowing synergistic action. However, challenges, such as large-scale production, the stability of nanoformulations, and the need for standardization, need to be considered, addressed, and explored.

### 6.5. Tetramethylpyrazine, Resveratrol and Curcumin (TRC)

**Figure 6:**
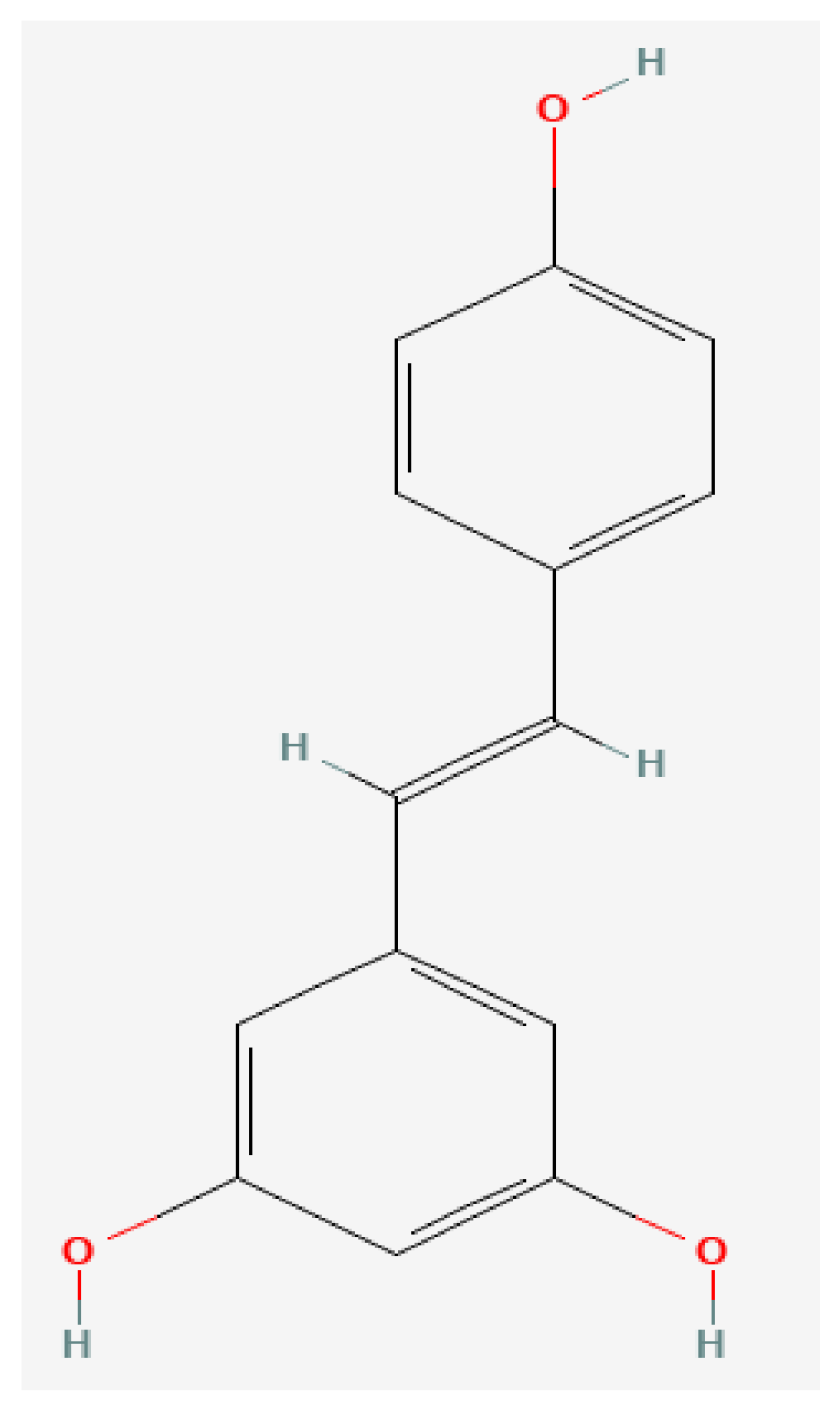
National Center for Biotechnology Information. PubChem Compound Summary for CID 445154, Resveratrol. https://pubchem.ncbi.nlm.nih.gov/compound/Resveratrol (accessed on 27 June 2021).

Resveratrol (Res) (Figure 6), a white powder commonly recognized for its ability to promote anti-inflammatory functions, can be found in greater proportions in grapes and red wine. Curcumin (Cur) (Figure 3) originates from the plant *Curcuma longa Linn*. and is known to be a yellowish compound. These natural compounds are flavonoids. Anti-inflammatory activities and, according to previous studies, minimal side effects are associated with RA treatment [66].

**Figure 7:**
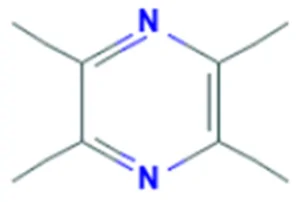
National Center for Biotechnology Information. PubChem Compound Summary for CID 14296, 2,3,5,6-Tetramethylpyrazine. https://pubchem.ncbi.nlm.nih.gov/compound/2_3_5_6-Tetramethylpyrazine (accessed on 27 June 2021).

Tetramethylpyrazine (TMP) (Figure 7), a compound obtained from *Ligusticum wallichi Franchat* (chuanxiong) from the Umbelliferae family, is commonly used to treat cardiovascular diseases and inflammatory diseases in China. According to Chen (2017) [66], the combination of flavonoids with TMP could be a possible therapeutic strategy for treating RA.

These compounds (TMP, Cur, and Res) were used in the same proportions to evaluate the following parameters: paw swelling volume, arthritis score, serum mediator concentration, histological examination and immunohistochemical staining [66].

Treatment with the compounds reduced the symptoms of arthritis compared with those in the control group. This combination of compounds represents a potential new therapeutic method to alleviate the symptoms of RA [66].

Tetramethylpyrazine is commonly used in China for the treatment of cerebrovascular and cardiovascular diseases. This compound is typically found in the form of pills and capsules. Owing to its wide use, some pharmacological parameters are found in the literature. Tetramethylpyrazine has a high first-pass effect and is rapidly eliminated, with a half-life between 0.8 and 2.8 h and an oral bioavailability of 10–30% in humans. This compound has a low molecular weight (136.2) and a low melting point, ranging from 76–78 °C, and an adequate solubility of 11 mg/mL [66].

Chen et al. (2017) [66] constructed a new transdermal system of the reservoir type using tetramethylpyrazine, in which they compared this procedure with the commonly used method, oral tablets. In this study, twenty male subjects with a mean age of 23 years were included. The main inclusion criterion was routine laboratory tests, which revealed good health. Single- and multiple-dose medications showed that the effect of the tetramethylpyrazine patch was comparable to that of oral medication. Additionally, the tetramethylpyrazine patch is associated with better patient compliance, fewer changes in drug dosage, and fewer adverse effects.

Amiot et al. (2013) [67] developed a new way to administer resveratrol in clinical treatments. In this study, fifteen healthy volunteers (men and women) received a new formulation of the substance resveratrol, namely, the soluble formulation (caplets) and the original powder (capsules). Patients received a single dose of 40 mg of resveratrol, and after this step, blood samples were collected at 15 min, 30 min and every hour for 5 h. The authors reported that the single administered dose of soluble resveratrol was well absorbed and remained at biologically efficient blood levels. The increase in bioavailability allowed a decrease in dosages commonly reported in the literature, and according to this study, no toxicity was reported. This study is fundamental for studying a new form of resveratrol that could improve the low absorption described in previous studies.

Azachi et al. (2014) [68] used red grape cells to assess their effects on oxidation and inflammatory stress. In this study, the authors analyzed the contents present in grape cells and detected polyphenols, with resveratrol being the major compound. In the first experiment, they used human blood to assess the effects of diet or pharmacological antioxidants on LDL oxidation. In the second experiment, the anti-inflammatory effects of red blood cells were evaluated in mice. Transresveratrol caused a decrease in carrageenan-induced hyperalgesia, demonstrating an inhibitory effect on LDL oxidation and revealing high bioavailability. This study revealed the antioxidant and anti-inflammatory properties of resveratrol. However, preclinical and clinical studies using grape red cells are indispensable.

In 2016, Banaszewska and colleagues studied the effects of resveratrol on polycystic ovary syndrome. In this study, thirty-four subjects were initially used, but thirty completed the experiment within three months. These subjects were divided into two groups: In the first group, they received 1500 mg of resveratrol powder; in the second group, they received a placebo. They reported a reduction in the serum levels of T and the hormone dehydroepiandrosterone in patients with polycystic ovary syndrome, showing effects on ovarian and adrenal androgens [69].

Sattarinezhad et al. (2018) [70] used resveratrol to reduce albuminuria in diabetic nephropathy. In this study, 60 patients with type 2 diabetes were treated with 500 mg of resveratrol per day in the first group and placebo in the second group for a period of 90 days. After this time of drug administration, resveratrol proved to be effective as an adjuvant angiotensin receptor blocker, reducing urinary albumin excretion in patients with diabetic nephropathy.

### 6.6. Melittin

Apitoxin bee venom, in its pure form, has been described as a potent mediator of anti-inflammatory, antiarthritic and neuroprotective effects. Additionally, melittin, which is a component of apitoxin bee venom, exerts anticancer effects. This venom is a complex mixture of biologically active peptides such as melittin or apamin; mast cell degranulation peptide; adolapine; amines such as histamine, dopamine and noradrenaline; enzymes such as phospholipase A2 and B; hyaluronidase; and various carbohydrates and lipids. Many of these peptides target ion channels and receptors in the peripheral and central nervous systems [71].

**Figure 8:**
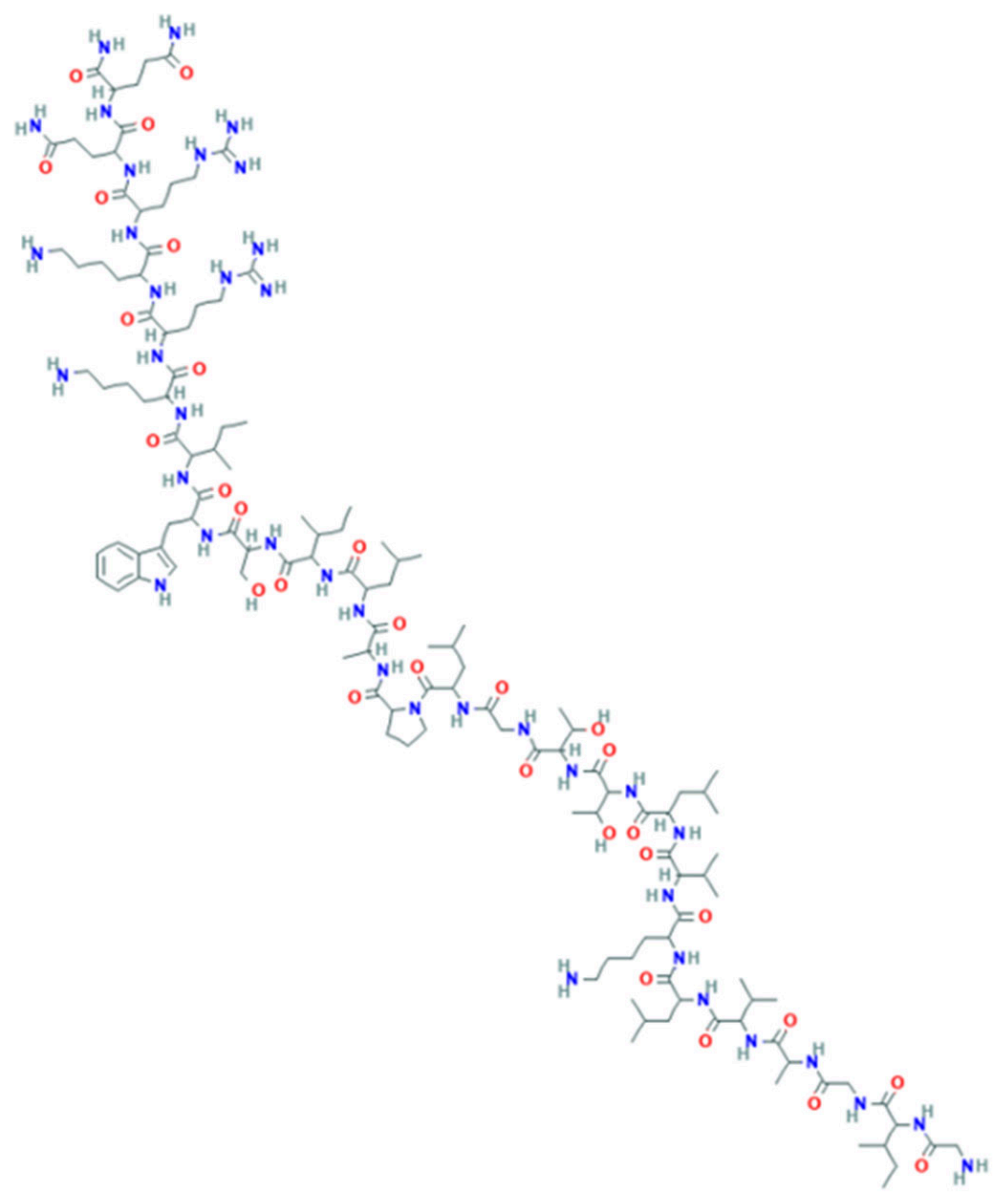
National Center for Biotechnology Information. PubChem Compound Summary for CID 16129627, Melittin. https://pubchem.ncbi.nlm.nih.gov/compound/Melitten (accessed on 5 July 2021).

In studies of rheumatoid arthritis, bee venom, especially melittin (Figure 8), has demonstrated anti-inflammatory effects. In an experimental murine model (*SD rats*, male, adult) with collagen-induced arthritis (CIA), bee venom has antinociceptive effects when it is injected into the Zusanli acupuncture point (ST36) at a dose of 0.25 mg/kg. They reported a change in tail movement latency, with the maximum analgesic effect observed 30 min after the start of bee venom injection and maintained for at least 60 min, whereas saline treatment, as well as apitoxin treatment without acupuncture, had no effect [72].

Furthermore, apitoxin inhibited the production of cytokines, such as IFN-γ, IL-1β, and TNF-α, in a CIA mouse model at high doses and further slowed disease progression in a dose-dependent manner. Additionally, the authors described a reduction in paw edema and radiological alterations, IL-6 levels and nociceptive behavior. Positive effects of apitoxin/melittin on RA were observed in cell culture using synoviocytes extracted from patients, with the suppression of IκKα and IκKβ activity by melittin and bee venom. Currently, IκK inhibitors are used for the treatment of inflammatory diseases, as they block the release of IκB, preventing the activity of NF-κB. Thus, the anti-inflammatory effects of melittin and bee venom in general highlight the potential of these natural compounds to alleviate RA symptoms [72,73].

Melittin inhibits the replication of several viruses, including HIV-1, by suppressing viral gene expression. Hartmann and colleagues evaluated the clinical efficacy and toxicity of melittin as a treatment for cats infected with feline immunodeficiency virus (FIV). In this study, 20 cats were naturally infected with FIV, and 10 were treated with 500 µg/kg melittin or phosphate-buffered saline (PBS) subcutaneously twice weekly for a period of 6 weeks. The overall health status and clinical scores for conjunctivitis and stomatitis improved in cats treated with melittin, and no local or systemic side effects were observed. However, this improvement was statistically significant for conjunctivitis in cats treated with melittin compared with that in cats treated with placebo. The use of higher doses for a longer period can be evaluated in further studies, as can the synergistic effects of coadministration with classic antiretroviral drugs [74].

Current conventional treatments are associated with significant toxicity, such as kidney damage and gastrointestinal bleeding, and investigating new drugs with therapeutic potential is essential. Melittin is a peptide extracted from bee venom that has been described as promising for the treatment of rheumatoid arthritis; however, it has adverse effects, such as allergic reactions, hemolysis, and cytotoxicity. Huang et al. investigated strategies described in the literature that improved the use of melittin as a therapy for rheumatoid arthritis. They observed that melittin was used as an acupuncture injection, preventing bone erosion in mice with collagen-induced RA (CIA). Another alternative is the use of melittin polymer microneedles, which, in addition to interacting with the weakly acidic microenvironment, regulate the release of encapsulated drugs. Furthermore, they reported in in vivo tests that the use of nanoparticles to better target the drug effectively reduced the side effects caused by melittin [75].

Melittin acts on multiple central targets of rheumatoid arthritis (RA), inhibiting the NF-κB pathway, reducing proinflammatory cytokines, and inhibiting metalloproteinases, which decreases synovial inflammation and type II collagen degradation and protects the joint. In this article, Pareek and colleagues discuss advanced melittin delivery systems that increase its effectiveness but also address how molecular modification, whether an alteration in the amino acid sequence or conjugation with other systems, can reduce toxicity and maintain its therapeutic effect [76].

### 6.7. Celastrol

**Figure 9:**
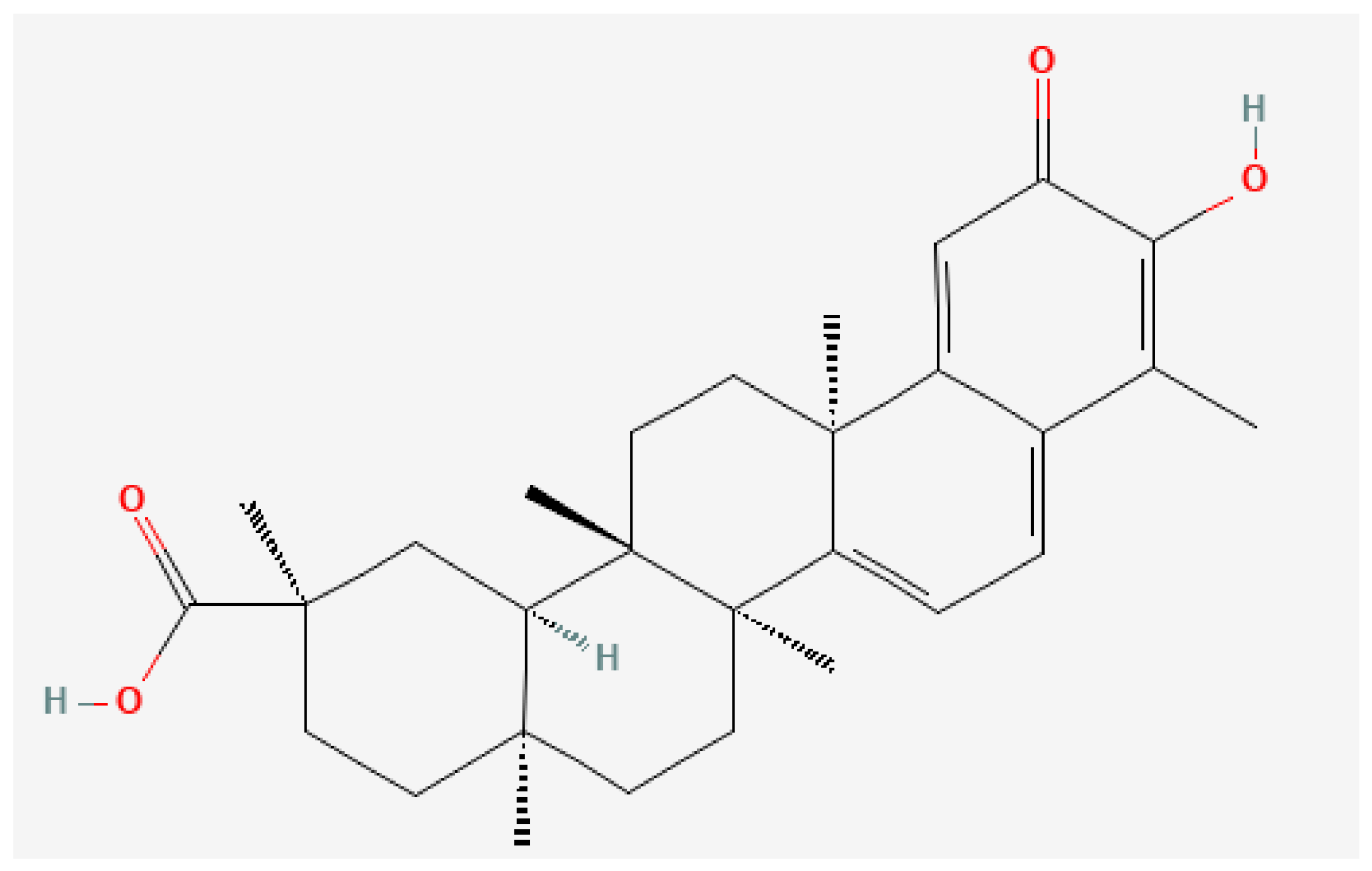
National Center for Biotechnology Information. PubChem Compound Summary for CID 122724, Celastrol. https://pubchem.ncbi.nlm.nih.gov/compound/Celastrol (accessed on 5 July 2021).

Celastrol (Figure 9) is a bioactive compound derived from traditional Chinese medicinal herbs of the Celastraceae family. Venkatesha et al. (2016) [77] demonstrated that celastrol acts on several molecular targets, such as the NF-κB pathway, which blocks the activity of I kappa B (IκB) kinase (IKK), as well as the degradation and phosphorylation of IκB; the MAPK pathway inhibits c-Jun N-terminal kinase (JNK) and extracellular signal-regulated kinase (ERK) phosphorylation; and the JAK/STAT pathway inhibits STAT3 phosphorylation and STAT3-mediated expression of extracellular signal-regulated kinase (ERK). Additionally, celastrol inhibits T helper 17 (Th17) differentiation and cell proliferation and the activity of the RANKL/OPG pathway, which is the key regulator of bone remodeling [77].

A review described the beneficial effects of celastrol in an adjuvant and collagen-induced arthritis model. Celastrol was able to reduce the levels of proinflammatory cytokines, as measured by lymph node cell drainage (LNC) and synovial cell infiltration (SIC), in arthritic Lewis rats treated with celastrol (3 g/kg) compared with control rats. Moreover, celastrol inhibited the expression and reduction in the amount of IL-17 in the supernatant of LNCs and SICs, respectively [77].

Celastrol also regulates the expression of chemokines and adhesion molecules and cell migration, which are elevated in the serum, synovial fluid, and synovial tissue of arthritis patients, leading to inflammation, pannus formation and local tissue damage. Furthermore, celastrol inhibits osteoclast activity and protects against joint damage [77].

Thus, celastrol is a promising candidate for further evaluation in patients with RA. Therefore, this compound may serve as a useful complement/alternative to conventionally used drugs whose long-term use is associated with serious adverse effects.

Celastrol is also known to alleviate cardiovascular symptoms, including hypertension. In a clinical study, celastrol was evaluated for the treatment of preeclampsia in combination with the antihypertensive drug nifedipine. A total of 626 patients aged between 22 and 35 years with preeclampsia were enrolled, selected, and randomly assigned to groups that received nifedipine + placebo or nifedipine + celastrol orally 15 min before blood pressure was measured. Analysis of the time to control hypertension revealed a significant reduction in the nifedipine + celastrol group (34.7 ± 21.0 min, *p* = 0.003), whereas the time to control pressure in the nifedipine + placebo group was 55.6 ± 16.2 min. The time before a new hypertensive crisis was prolonged with treatment (7.9 ± 1.7 h) compared with that in the nifedipine + placebo group (5.3 ± 1.9 h, *p* = 0.002). Furthermore, the number of doses needed to control hypertension was reduced in the nifedipine celastrol group [78].

### 6.8. Kaempferol

**Figure 10:**
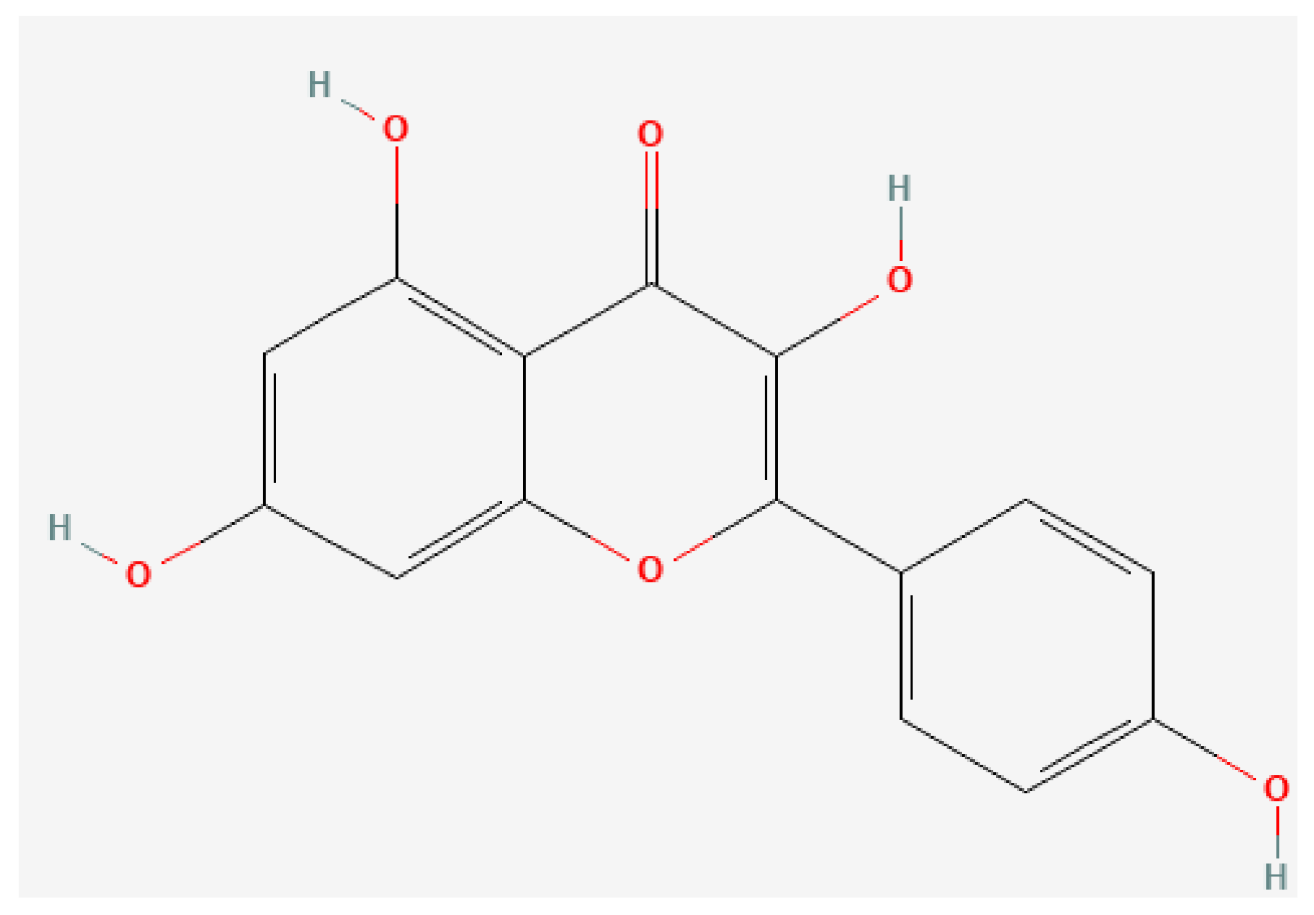
National Center for Biotechnology Information. PubChem Compound Summary for CID 5280863, Kaempferol. https://pubchem.ncbi.nlm.nih.gov/compound/Kaempferol (accessed on 5 July 2021).

Kaempferol (Figure 10) is a natural flavonol present in different plant species and has been described as having potent anti-inflammatory properties. Several groups have demonstrated that kaempferol inhibits LOX, which is an enzyme responsible for the formation of leukotrienes (LTs) from the arachidonic acid (AA) pathway and in various inflammatory disorders, including asthma, RA, and inflammatory bowel disease, both in vitro and in vivo [79].

Treatment of murine microglial BV2 cells and RA synovial fibroblasts (10–200 µM) with kaempferol significantly inhibited LPS- and IL-1β-induced JNK and p38 phosphorylation, nitric oxide production, PGE2 expression and iNOS expression. LPS-induced lung injury in BALB/c mice has been shown to activate ERK, p38 and JNK phosphorylation and thus mediate the expression of proinflammatory mediators, ROS, and myeloperoxidase (MPO). However, kaempferol treatment suppressed the activation of the ERK, p38 and JNK pathways and alleviated their harmful effects [79].

An imbalance between matrix metalloproteinases (MMPs) and tissue inhibitors of metalloproteinases results in several pathological conditions, including inflammation. Proinflammatory cytokines, including IL-1, TNF-α, and IFN-γ, have been reported to stimulate matrix metalloproteinase expression through NF-κB activation. Yoon et al. reported an increase in MMP expression at both the mRNA and protein levels with IL-1 treatment and a significant reduction in kaempferol supplementation in RA synovial fibroblasts [79,80].

A prospective study evaluated the associations between the intake of flavonoids, including kaempferol, and the risk of total and specific cancers in middle-aged women via a validated 131-item semiquantitative food frequency questionnaire. However, no significant associations were detected between the initial intake of 5 common flavonoids or flavonoid-rich foods and cancer prevention [81].

Another study evaluated the potential independent and additive effects of daily supplementation with alpha-linolenic acid (ALA) (3.6 g) and quercetin (190 mg) for an 8-week period on blood pressure and lipid and glucose metabolism, as well as biomarkers of inflammation, oxidative stress, and antioxidant status, in healthy nonobese (n = 67) males and females. Compared with the control treatment, the ALA + quercetin treatment, but not the ALA + placebo treatment, significantly increased the plasma quercetin, tamoxifen, isorhamnetin and kaempferol concentrations at baseline and at the end of the study. The authors described significant improvement in lipid profiles. However, no evidence of an additive or synergistic effect of ALA plus quercetin on cardiovascular disease risk markers has been reported [82].

In the systematic review produced by Nazir and colleagues, the therapeutic effects of kaempferol in arthritis (including RA) were systematically and quantitatively analyzed on the basis of preclinical, in vitro, and in vivo models. They concluded that kaempferol reduced histological damage, joint edema, and oxidative stress and reduced TNF-α, IL-1β, IL-6, and IL-17 levels; thus, kaempferol is considered a promising phytotherapeutic for treating arthritis, especially RA [83].

Hong et al. (2025) [84] reviewed the potential of kaempferol and highlighted its immunomodulatory effect, especially on T lymphocytes, in addition to increasing the activity of enzymes such as superoxide dismutase (SOD) and glutathione (GSH), which are antioxidants. Furthermore, they reported that kaempferol can regulate cellular processes by inducing the apoptosis of inflammatory cells and reducing the proliferation of synovial fibroblasts. However, the predominance of preclinical evidence still limits its clinical application.

### 6.9. Hesperidine

**Figure 11:**
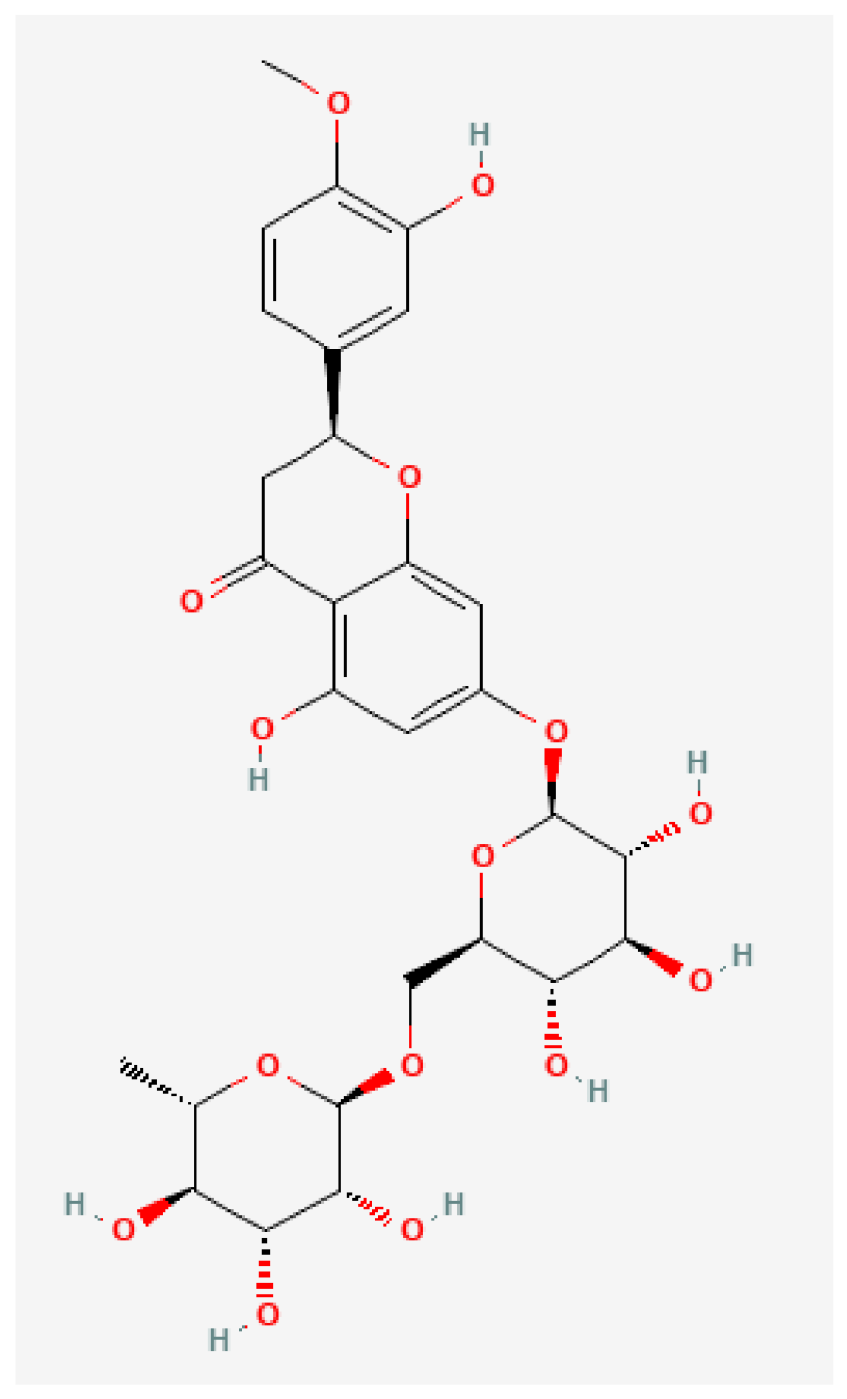
National Center for Biotechnology Information. PubChem Compound Summary for CID 10621, Hesperidin. https://pubchem.ncbi.nlm.nih.gov/compound/Hesperidin (accessed on 5 July 2021).

Hesperidin (HES) (Figure 11), a natural flavonoid from citrus fruits and vegetables, has a range of pharmacological effects, including immunomodulatory, anti-inflammatory, antioxidant, and antiapoptotic effects. A study in CIA mice revealed that HES treatment substantially decreased clinical scores and improved histological features. The administration of HES in a complete Freund’s adjuvant (CFA)-induced murine model of RA considerably increased the serum levels of all biomarkers (COMP, IgG, ANA, MPO, MDA, and GSH) to normal levels [85].

To further explore the mechanisms underlying the therapeutic effects of HES, HES was administered intragastrically to rats with adjuvant-induced arthritis. The results revealed that HES dose-dependently alleviated secondary swelling of the paw, decreased the rate of polyarthritis, and attenuated pathological changes, possibly through the downregulation of overactivated peritoneal macrophages and the upregulation of dysfunctional T lymphocytes, which subsequently modulated the secretion of IL-1, IL-6 and TNF-α and the production of IL-2. Furthermore, daily treatment with HES at a dosage of 160 mg/kg body weight for CIA rats from disease onset until day 20 reduced all parameters and decreased the levels of IL-1, IL-6 and TNF-α. These data further revealed an improvement from HES to CIA, as measured by the release of free radicals by activated neutrophils and several other biochemical pathways, as well as a reduction in neutrophil infiltration into the joints [86].

In LPS-induced fibroblast-like synovial cells (FLSs), HES treatment has significant anti-inflammatory effects, reducing the potential of metalloproteinases in RA and inhibiting macrophage polarization to the M1 phenotype in a mouse model of antigen-induced arthritis [86]. These results suggest that the administration of hesperidin may be effective for the treatment of human patients with RA.

The use of HES has been reported in clinical trials for several diseases. In a clinical study, supplementation with 1 g of HES for 12 weeks in 50 patients with nonalcoholic fatty liver disease (NAFLD) inhibited several characteristic parameters of NAFLD, such as alanine aminotransferase (*p* = 0.005), γ-glutamyltransferase (*p* = 0.004), total cholesterol (*p* = 0.016), triglycerides (*p* = 0.049), hepatic steatosis (*p* = 0.041), high-sensitivity C-reactive protein (*p* = 0.029), tumor necrosis factor-α and nuclear factor-κB (NF-κB) [87].

Cheraghpour and collaborators reported a study evaluating the effectiveness of HES in normalizing the set of risk factors in 49 patients with metabolic syndrome. HES supplementation (500 mg) twice daily for 12 weeks decreased fasting glucose levels (−6.07 vs. −13.32 mg/dL; *p* = 0.043), triglyceride levels (−8.83 vs. −49.09 mg/dL; *p* = 0.049), and systolic blood pressure (−0.58 vs. −2.68 mmHg; *p* = 0.048) and TNF-α levels (−1.29 vs. −4.44 pg/mL; *p* = 0.009), whereas in the control group, only glucose and insulin levels decreased significantly, indicating that hesperidin supplementation may improve metabolic abnormalities and the inflammatory status in patients with metabolic syndrome [87].

In another clinical study, HES combined with plasminogen activator (rt-PA) for 7 consecutive days diminished adverse symptomatic intracerebral hemorrhage in patients with ischemic stroke [88]. For RA treatment, Kometani et al. (2008) [89] evaluated the effect of alpha-glucosishesperidine (HES-G), a derivative of HES with greater solubility in water. In the double-blind trial, 3 of the 9 patients who received HES-G treatment improved, whereas only 1 of the 10 patients in the placebo group improved, which was likely due to standard therapy with a physician that all patients underwent every 4 weeks [89]. A flavonoid mixture containing hesperidin (150 mg), diosmin (150 mg), troxerutin (300 mg), rutin (250 mg) and quercetin (150 mg) was found to be a safe and effective means of controlling bleeding from hemorrhoidal disease, with few adverse events reported in a study of 154 patients with hemorrhoidal disease [90].

In addition to its diverse medicinal properties, HES also exerts hepatoprotective effects on a variety of natural and chemical hepatotoxins through different mechanisms, such as increased nuclear factor 2/antioxidant response element and heme oxygenase 1 activities, as well as increased levels of enzymatic and nonenzymatic antioxidants. Other important hepatoprotective mechanisms of hesperidin include a reduction in the levels of high mobility group box 1 protein, alpha kappa B protein inhibitor, matrix metalloproteinase-9 and C-reactive protein [91].

Long et al. (2023) [92] conducted a systematic review and meta-analysis of 47 randomized clinical trials to evaluate the efficacy and safety of dietary polyphenols in rheumatoid arthritis. Polyphenols are water-soluble compounds derived from plants that have antioxidant, anti-inflammatory, and immunomodulatory properties. Among the polyphenols studied, hesperidin improved RA symptoms in three out of nine patients (compared with the control group, in which only one out of ten patients improved). Hesperidin inhibited free radical scavenging, COX-2 expression, macrophage polarization via the PI3K/AKT pathway, and synoviocyte proliferation.

## 7. Essential Oil

### Copaiba Oil

Copaiba oil is an herbal medicine used to treat arthritis [11]. This compound is a natural product widely used in Amazonian folk medicine because of its various therapeutic effects, low cost, and easy access [17]. Copaiba is a native Brazilian tree of the genus *Copaifera* sp. belonging to the botanical family Leguminosae. Abundant in the Amazon region, its oil is produced by exuding the trunks of trees of this genus. In 1972, copaiba was approved as an herbal medicine by the Food and Drug Administration (FDA), with more than 30 ethnopharmacological indications described in the literature [93,94].

Studies in rats with arthritis have shown that the anti-inflammatory action of this compound, at doses of 0.58 and 1.15 g/kg, was partially effective despite having reduced paw edema (0.1 mL (500 µg) of Freund’s adjuvant). Copaiba was not able to minimize secondary lesions due to arthritis in the tail or ears. The observed toxic effects vary according to the dose used [11].

Despite the proven effectiveness of some properties, studies with copaiba oil revealed cytological changes in various organs, such as the kidneys, lungs, and liver, suggesting a duality in oil performance [17]. The dose of 1150 mg/kg copaiba oil was toxic to the liver. In contrast to previous studies on the toxic dose of this herbal medicine, which suggested that the toxicity of this oil is greater than 2.000 mg/kg, lower doses would be safe for therapeutic use [11].

The discrepancy in the studies on the toxic dose of copaiba oil can be explained by differences in the criteria for estimating the acute toxicity (LD_50_, median lethal dose) of copaiba oil-resin. In general, they chose only behavioral changes or death as evaluation criteria for the animals studied [11].

Copaiba oil consists of two classes of secondary metabolites, namely, sesquiterpenes and acidic diterpenes. The main sesquiterpenes found are β-elemene and β-caryophyllene (BCP), which constitute the volatile fraction, and the main diterpenes are ent-hardwickiic acid and ent-copalic acid, which compose the fixed fraction [95,96].

Sesquiterpene β-caryophyllene, the major constituent of copaiba oil, is considered a compound of particular interest for the development of anti-inflammatory and antirheumatic drugs. This compound has a cyclobutane ring and a trans double bond in a nine-carbon ring in its structure, both of which are rare in nature [96,97]. This compound has low water solubility and consequently low absorption by the cell. However, BCP has been shown to interact with artificial lipid bilayers, suggesting a high affinity for the cell membrane [97].

Arthritic and healthy rats were treated for 18 days with copaiba essential oil (CEO), either in its nonformulated form or incorporated into a self-nanoemulsifying drug delivery system (FSNEDDS), at doses of 50 and 100 mg/kg. At 50 mg/kg, nonformulated CEO had limited effects, reducing only the weight of popliteal lymph nodes. At 100 mg/kg, CEO decreased total leukocyte counts and liver myeloperoxidase activity; however, it did not significantly affect other parameters, such as the arthritis score or paw edema. Importantly, nonformulated CEO did not induce hepatotoxicity at either dose in healthy rats, as evidenced by unchanged hepatic gluconeogenesis and stable plasma biochemical markers. Nevertheless, morphometric analysis revealed a reduction in the number of hepatocytes per unit area, along with an increase in hepatocyte size [98].

In contrast, FSNEDDS exhibited pronounced antiarthritic activity, particularly at the 100 mg/kg dose. Notably, these effects were not associated with overt hepatotoxicity, despite the morphometric changes observed in the liver of arthritic animals, including a decrease in the area of hepatocytes and an increase in the number of hepatocytes. Additionally, treatment with FSNEDDS at both doses promoted weight gain in arthritic rats. This finding is relevant in the context of rheumatoid cachexia, a condition that typically impairs weight gain, suggesting a potential beneficial metabolic effect. With respect to oxidative stress, FSNEDDS reduced the levels of carbonyl protein groups in both the liver and plasma, whereas this effect was not observed with the nonformulated CEO. Furthermore, FSNEDDS demonstrated superior anti-inflammatory activity at a dose of 100 mg/kg, improving the endogenous antioxidant defense system and contributing to the normalization of reactive oxygen species (ROS) levels in the liver [98].

Among the substances mentioned above, some are found in commercial form, such as *Salix alba*, which is found in the form of tablets. According to the literature, this medication may have several side effects, such as headache, drowsiness, fever, myalgia [99] and, in some cases, nephrotoxicity [12]. Some of the substances found do not cause a high level of toxicity. However, they depend on the extent of the treatment to be used [99].

*Curcuma longa* can be found in capsule form. This species has adverse effects that are considered low but has drug interactions with anticoagulants [100] and NSAIDs, in addition to having a high cost.

*Cannabis sativa* is a promising species for treating RA. However, as *C. sativa* is considered an illicit narcotic, the derived products make treatment extremely expensive (CRF-RS, 2015) because of customs fees and bureaucratic processes [101].

Pomegranates and propolis are found commercially and have easy access to the population. These products are relatively inexpensive. However, they have adverse effects, such as blood pressure changes [30].

From the searches, we were not able to find commercial versions of the other substances mentioned during this study. Some of them should be manipulated concerning their components but with difficult access to the drug.

## 8. Computational Analysis of Isolated Substances—Results and Discussion

### 8.1. Physical—Chemical Properties

The physicochemical properties of compounds have been used to estimate their behavior in biological environments. The concept of drug likeness defines the physicochemical characteristics of a molecule as a potential drug [102]. In this context, Lipinski’s rule of five was developed to evaluate the drug likeness of a drug to become an orally active drug in humans. Thus, we explored Lipinski’s rule for compounds with the potential to treat RA. Table 1 presents the overall physicochemical results.

**Table 1:** Physicochemical parameters of the compounds compared with those of commercial anti-inflammatory drugs (diclofenac, ibuprofen, and naproxen). ^a^ Molecular weight (Da) (100~600); ^b^ Partition coefficient on a logarithmic scale (the logarithm of n-octanol/water distribution coefficients at pH = 7.4); ^c^ Native water solubility (mg/mL) (the logarithm of the aqueous solubility value. LogS = log_10_ (molar solubility in mol/L) Ideal: Compounds with LogS in the range of −4 to 0.5 log mol/L are considered suitable. Low Solubility: Very low values (generally less than −4) indicate poor solubility, which can impair oral absorption); ^d^ Number of hydrogen bond acceptors (HBAs) (Optimal:0~12); ^e^ Number of hydrogen bond donors (HBDs) (Optimal:0~7); ^f^ Number of Lipinski’s rules broken (NLRB) (MW ≤ 500; logP ≤ 5; Hacc ≤ 10; Hdon ≤ 5). ^g^ Lipinski’s violation code: LP-lipophilicity, HBA—hydrogen bond acceptor, MW—molecular weight, HBD—hydrogen bond donor (whose properties were outside the range during the analyses. Representative of the symbology of each toxicity analysis).

Compounds	Physical-Chemical Properties						
	^a^ MW	^b^ logP	^c^ logS	^d^ HBA	^e^ HBD	^f^ NLRB	^g^ NLRB Code
Cannabidiol	314.471	6.382	0.016	2	2	0	LP
Curcumin	368.389	2.994	0.045	6	2	0	-
Paeoniflorin	480.472	0.046	1.333	11	5	1	HBA
Quercetin	302.242	1.958	0.044	7	5	0	-
Tetrametilpirazina	136.198	1.565	3.708	2	0	0	-
Resveratrol	228.249	3.094	0.169	3	3	0	-
Melittin	2846.522	−15.675	6.455	69	41	3	HBA; HBD Mw;
Celastrol	450.622	5.766	0.002	4	2	1	LP
Kaempferol	286.243	2.243	0.125	6	4	0	-
Hesperidine	610.573	−0.327	4.894	15	8	3	HBA; HBD; MW;
diclofenac	295.155	4.434	0.0464	3	2	0	-
Ibuprofen	206.287	3.646	0.1	2	1	0	-
Naproxen	230.265	3.21	0.0649	3	1	0	-

The compounds curcumin, quercetin, tetramethylpyrazine, resveratrol, and kaempferol did not violate Lipinski’s rule, suggesting the high potential of these compounds as orally active drugs in humans. On the other hand, the compounds melittin and hesperidine presented poor drug likeness profiles, as they presented three violations of Lipinski’s rule.

### 8.2. Pharmacokinetics Parameters

The evaluation of the pharmacokinetic parameters indicated that the compounds have a poor ability to cross the blood–brain barrier, except for cannabidiol and tetrametilpirazin. A comparison of jejunal permeability with that of commercial anti-inflammatory drugs revealed that the compounds cannabidiol, curcumin, tetrametilpirazina, and celastrol presented similar profiles. Additionally, the evaluated compounds are usually not predicted to be substrates for P-glycoprotein, except for paeoniflorin, melittin, celastrol, and hesperidine, suggesting a low permeability for these compounds.

In addition, compared with some commercial anti-inflammatory drugs, some compounds, such as paeoniflorin, tetrametilpirazina, melittin, and hesperidine, present a considerably larger fraction of unbound plasma protein. Unbound drugs in plasma can exhibit pharmacological activity by interacting with targets, and the unbound fraction in the plasma of a drug is an important factor in determining drug efficacy [102]. Only tetrametilpirazina presented a higher blood-to-plasma concentration (greater than one), suggesting that red blood cells are predicted to sequester tetrametilpirazina, leading to a lower free circulating fraction of this compound.

The volume distribution (V_d_) is a pharmacokinetic value that is directly proportional to the amount of drug distributed in the tissue. The compounds quercetin, resveratrol, melittin, celastrol, and kaempferol presented Vd profiles similar to those of commercial anti-inflammatory drugs. On the other hand, the compounds cannabidiol, paeoniflorin, and tetrametilpirazina presented higher V_d_ values, suggesting a greater tissue distribution for these compounds.

Finally, the ADMET risk indicates that the compounds curcumin, tetrametilpirazin, and resveratrol present high potential for successful development as drugs, with values similar to or even smaller than those of commercial anti-inflammatory drugs. Table 2 presents the pharmacokinetic parameters of these compounds.

**Table 2:** Pharmacokinetic parameters of the compounds compared with those of commercial anti-inflammatory drugs (diclofenac, ibuprofen, and naproxen); ^a^ Qualitative likelihood (High/Low) of crossing the blood–brain barrier (BBB) (Category 1: BBB+; Category 0: BBB−; The output value is the probability of being BBB+. The outputs are classified as high, medium, or low.); ^b^ Human jejunal effective permeability (cm/s × 10^4^) (Peff) (molecules with log Peff values less than 2.0 were classified as low-permeability (Category 0), while those with log Peff values exceeding 2.5 were classified as high-permeability (Category 1)); ^c^ Fu: The fraction unbound in plasms (Low: <5%; middle: 5~20%; high: >20%) In pharmacology and medicinal chemistry, this parameter refers to the percentage of a drug that circulates “free” and active in the blood, unbound to plasma proteins. This classification indicates the compound’s bioavailability and potential activity.; ^d^ Blood–plasma concentration ratio in humans (RBP) (plasma protein binding optimal: <90%.); ^e^ Pharmacokinetic volume of distribution in humans (L/kg) (V_d_) (optimal: 0.4–20 L/kg.); ^f^ Likelihood of P-glycoprotein efflux (P-gp); ^g^ ADMET_Risk calculates the risk of potential obstacles to a compound being successfully developed as an orally bioavailable drug.

Compounds	Pharmacokinetics Parameters						
	^a^ BBB	^b^ Peff	^c^ Fu	^d^ RBP	^e^ Vd	^f^ P-gp	^g^ ADMET_Risk
Cannabidiol	High	6.332	4.601	0.747	1.47	No	4.565
Curcumin	Low	5.149	6.086	0.758	0.80	No	0.057
Paeoniflorin	Low	0.215	49.554	0.68	1.28	Yes	7.539
Quercetin	Low	2.051	12.063	0.82	0.32	No	3.914
Tetrametilpirazina	High	7.752	65.645	1.12	1.42	No	1
Resveratrol	Low	3.752	7.53	0.883	0.56	No	1.032
Melittin	Low	0.005	61.792	0.704	0.23	Yes	8.082
Celastrol	Low	6.253	5.229	0.722	0.30	Yes	4.856
Kaempferol	Low	2.311	9.84	0.801	0.33	No	2.433
Hesperidine	Low	0.128	25.782	0.733	0.79	Yes	7
Diclofenac	High	6.40	3.726	0.672	0.26	No	1.004
Ibuprofen	High	6.52	5.861	0.704	0.34	No	0.031
Naproxen	High	6.44	5.336	0.694	0.23	No	0.951

### 8.3. Toxicological Profile

Cardiovascular diseases are among the leading causes of morbidity and mortality worldwide. Drugs can significantly contribute to enhancing cardiovascular risk factors and thus deserve special attention. The human ether-a-go-go-related gene (hERG) is a gene that encodes potassium channels, which mediate repolarization of the ion current in the cardiac action potential. Drug-induced blockade of potassium channels in heart cells can lead to life-threatening ventricular arrhythmias. Therefore, potassium channel blockade predictions were computed for the compounds, and none of them presented potential for channel blockade.

Drug-induced liver injury (DILI) is a well-recognized problem, with an estimated annual incidence of between 1.3 and 19.1 per 100,000 people exposed. DILI is also the most frequently cited reason for the withdrawal of medications from the marketplace (up to 32% of drug withdrawals) [103,104]. DILI is usually indicated if the serum levels of aspartic acid transaminase (AST) and alanine transaminase (ALT) are both elevated. Liver injury also usually increases serum levels of lactate dehydrogenase (LDH). Concomitant elevation of other serum enzymes, such as alkaline phosphatase (AlkPhos) or gamma-glutamyltransferase (GGT), is indicative of even more severe liver injury. Thus, we predicted that the potential of each of the five enzymes would be elevated. All the evaluated compounds presented at least one increase in enzyme activity. The compound cannabidiol is predicted to increase only LDH levels, suggesting a low risk of hepatotoxicity. On the other hand, the compound tetrametilpirazina is predicted to increase the levels of all the enzymes related to DILI, thus suggesting a high risk of hepatotoxicity. Importantly, the commercial drug diclofenac also has the potential to increase the activity of four enzymes; in fact, many works have ranked diclofenac as the leading cause of drug-induced liver injury caused by anti-inflammatory drugs [105,106].

All the evaluated compounds presented a low overall mutagenicity risk, and only quercetin and kaempferol presented slightly greater mutagenicity values than did naproxen did. On the other hand, some compounds, such as curcumin, quercetin, resveratrol, celastrol, and kaempferol, are predicted to trigger mutagenic chromosomal aberrations.

The potential of the compounds to cause reproductive toxicity was also evaluated, and only the compounds cannabidiol, curcumin, resveratrol, melittin, and kaempferol presented toxicity risks.

Finally, all the evaluated compounds presented a low overall toxicological risk profile, as indicated by the TOX_Risk parameter, which is a computational filter developed by Simulations Plus using a refined subset of WDI, which includes a diverse set of toxicological models. Additionally, the TOX_Risk values were similar to or lower for the evaluated compounds than for the commercial anti-inflammatory drugs. Table 3 presents the toxicological parameters of the compounds.

**Table 3:** Toxicological profile of the compounds in comparison with commercial anti-inflammatory drugs (diclofenac, ibuprofen and naproxen); ^a^ hERG (hERG potassium channel inhibition): Measures the likelihood of the molecule blocking the human hERG potassium channel. Blocking this channel is strongly associated with cardiotoxicity—specifically, QT interval prolongation on the electrocardiogram—which can lead to serious arrhythmias. Classified as Yes (blocker/cardiac risk) or No (non-blocker). Molecules with an IC50 ≤ 10 µM or ≥50% inhibition at 10 µM were classified as hERG+ (Category 1). Molecules with an IC50 > 10 µM or <50% inhibition at 10 µM were classified as hERG− (Category 0); ^b^ CABR (Induction of Mutagenic Chromosomal Aberrations): Qualitatively assesses whether the chemical substance has the potential to cause structural or numerical damage to the chromosomes of mammalian cells (Toxic or Nontoxic). ^c^ Hepatotoxicity: These are the values used to assess the Hepatotoxicity Profile (Liver Enzymes). The acronyms represent classic enzymes and biomarkers of injury or altered function in the human liver. ADMETlab evaluates the likelihood of the compound causing an elevation in each of them (Normal or Elevated). ALT (Alanine Aminotransferase/TGP): An enzyme highly specific to the liver. Elevated blood levels are a direct sign of hepatocyte injury (necrosis or hepatic inflammation). AST (Aspartate Aminotransferase/TGO): An enzyme present in the liver, but also in muscles and the heart. Elevated levels suggest cellular damage in the liver or other muscle tissues. LDH (Lactate Dehydrogenase): A widely distributed cytoplasmic enzyme; its elevation in plasma indicates generalized cell lysis (destruction) or acute tissue injury (including the liver). GGT (Gamma-Glutamyl Transferase): A sensitive marker for liver toxicity, bile duct injury, and oxidative stress in the liver. AlkPhos (Alkaline Phosphatase): An enzyme associated primarily with the cell membranes of bile ducts and bones; its elevation generally indicates cholestasis (interruption of bile flow) or biliary dysfunction; ^d^ Repro (Reproductive Toxicity): A qualitative estimate of whether the compound has the potential to negatively affect fertility (male or female) or cause developmental toxicity in offspring. Classified directly as Toxic or Nontoxic; ^e^ MUT_Risk (Mutagenicity Risk): Represents the mutagenicity risk assessed by ten integrated “virtual Ames test” models. Values greater than 0.5 indicate a high probability that the substance is Ames-positive (mutagenic); ^f^ TOX_Risk (Global Toxicological Risk): An index that calculates the overall accumulated toxicological risk of the molecule. A score of 0 represents zero or low risk; a score below 2 indicates a low-to-moderate risk considered acceptable; a score greater than or equal to 2 indicates high risk; and the maximum score of 7 represents extremely high risk across multiple parameters.

Compound	Toxicological Profile									
	^a^ hERG	^b^ CABR	^c^ Hepatotoxicity					^d^ Repro	^e^ MUT_Risk	^f^ TOX_Risk
			AST	ALT	LDH	GGT	AlkPhos			
Cannabidiol	No	Nontoxic	Normal	Normal	Elevated	Normal	Normal	Toxic	0	0
Curcumin	No	Toxic	Normal	Elevated	Normal	Elevated	Normal	Toxic	0.6	0
Paeoniflorin	No	Nontoxic	Elevated	Elevated	Normal	Normal	Elevated	Nontoxic	0.6	1.229
Quercetin	No	Toxic	Elevated	Normal	Elevated	Normal	Elevated	Nontoxic	1.8	1
Tetrametilpirazina	No	Nontoxic	Elevated	Elevated	Elevated	Elevated	Elevated	Nontoxic	0	1
Resveratrol	No	Toxic	Elevated	Elevated	Normal	Elevated	Elevated	Toxic	0.6	0
Melittin	No	Nontoxic	Normal	Elevated	Normal	Normal	Elevated	Toxic	0.6	2
Celastrol	No	Toxic	Elevated	Elevated	Elevated	Normal	Normal	Nontoxic	0	2
Kaempferol	No	Toxic	Elevated	Normal	Elevated	Elevated	Elevated	Toxic	1.8	1
Hesperidine	No	Nontoxic	Elevated	Normal	Normal	Normal	Elevated	Nontoxic	0.6	1
Diclofenac	No	Nontoxic	Elevated	Elevated	Normal	Elevated	Elevated	Nontoxic	0.6	2
Ibuprofen	No	Nontoxic	Normal	Normal	Normal	Elevated	Normal	Nontoxic	0.6	0
Naproxen	No	Toxic	Normal	Normal	Normal	Normal	Normal	Nontoxic	1.2	0

## 9. Target Fish

### Potential Molecular Targets Based on H. sapiens

**Figure 12:**
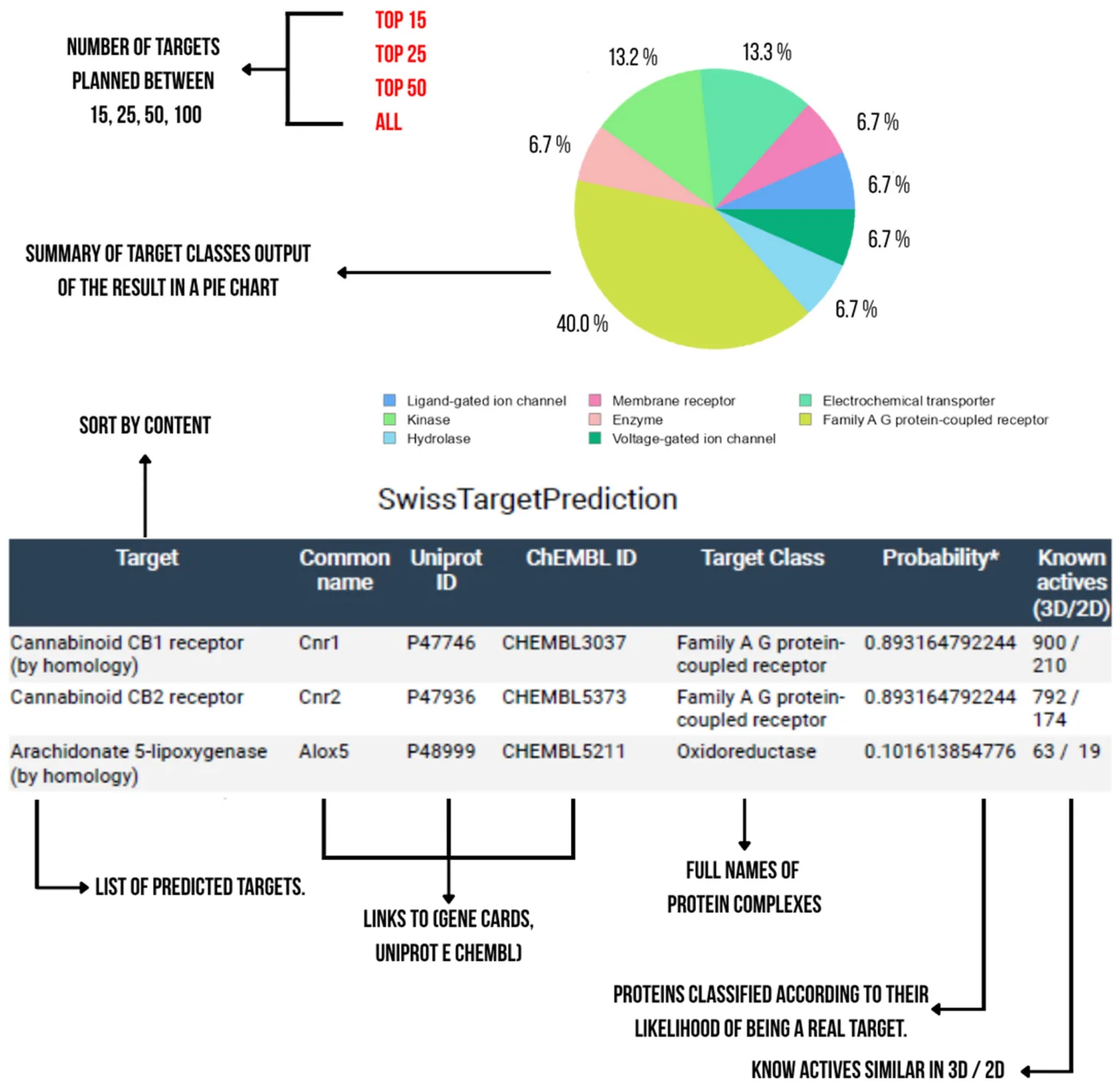
Target fishing prediction plot cannabidiol. Source: Adapted from SwissTargetPrediction [108], edited and graphically designed by the autor using Adobe Illustrator 2025 (v30.1). The results page displays the structure of the analyzed molecule; in this case, we used cannabidiol, obtained via the web server (https://commonchemistry.cas.org/detail?cas_rn=13956-29-1) retrieved 3 February 2026 (CAS RN: 13956-29-1). Licensed under the Attribution-Noncommercial 4.0 International License (CC BY-NC 4.0). In the top right corner, a summary of the predicted target classes is presented in a pie chart; percentages are calculated based on the top 15, 25, or 50 targets, or all predicted targets (up to a maximum of 100). The main table lists the predicted targets with the highest probability for the query molecule. This probability parameter is displayed in the column labeled *Probability*; the protocol ranks proteins by the likelihood of being a genuine target (indicated by green bars or numerical values), though the dynamic table can be sorted by any column by clicking the corresponding header. By default, the server displays 15 rows, but users can choose to view 15, 25, 50, or all predicted targets, up to a maximum of 100 results. Alongside the results, links to GeneCards, UniProt, and ChEMBL are provided. Proteins are listed by their full names, with individual links for each subunit found in the GeneCards and UniProt databases. The final column shows the number of known active compounds for each listed target that exhibit high similarity to the query molecule, based on 2D or 3D similarity metrics. These numbers serve as links redirecting to pages containing information about the similar molecules. This column allows users to download files containing known active compounds for the target that show 2D similarity to the query molecule. A separate file is also available containing all known active compounds for the same target that exhibit 3D structural similarity to the query molecule. Predictions derived from active molecules in homologous or orthologous proteins are labeled as such or indicated as being homology-based. Adapted from [108,109,110].

The virtual screening began with a search of approximately 3000 molecular targets of *Homo sapiens* allocated to a database on the Swiss Target Prediction online server (http://www.swisstargetprediction.ch/). The initial steps were carried out via the similarity search method, resulting in the prediction of 50 molecular target proteins of the compounds cannabidiol, curcumin, paeoniflorin, quercetin, tetramethylpyrazine, resveratrol, celastrol, kaempferol, and hesperidin. However, for each compound, the probabilities of interaction with these molecular targets were less than 0.5 according to their scoring function, and the 50 proteins with the best probabilities were selected for reverse docking analysis (Figure 12) [107].

After the reverse docking technique was applied, the 50 proteins identified in the previous step were analyzed to determine the most promising molecular targets capable of interacting with the compounds described in the previous paragraph of this study. On the basis of the probability scores obtained, the top ten targets for each compound were selected for further analysis. These results are summarized in Table 4, which lists the most relevant predicted protein targets for each substance.

The virtual screening applied via the target fishing technique involves approaches based on both ligands and receptors; more than 3000 molecular targets in *Homo sapiens* and 100 molecular targets in Cannabidiol, Curcumin, Paeoniflorin, Quercetin, Tetramethylpyrazine, Resveratrol, Celastrol, Kaempferol and Hesperidin have been evaluated, resulting in proteins identified as targets for future studies.

The next steps of this proposed study must continue to refine the results via molecular docking techniques and molecular dynamics simulations, which allow investigations of the most likely targets of the compounds of the isolated substances, cannabidiol, curcumin, paeoniflorin, quercetin, tetramethylpyrazine, resveratrol, celastrol, kaempferol and hesperidin, in greater atomic and molecular detail.

The method applied was target fishing with two approaches: similarity searching (ligand-based approach) and reverse docking (receptor-based approach) with the aid of the Swiss Drug Design web server [14].

The study was conducted in layers so that subsequent screening and good funneling of the data revealed the molecular targets with the greatest probability of interacting with the compounds we analyzed. The computational stage of the similarity search explored an approach in the database containing approximately 3000 mammalian molecular targets.

The molecules were sent to a web server (http://www.swisstargetprediction.ch/) one by one in SMILES on the basis of the selection of the target species of each protein so that the prediction could be made for our study target; the selected species was *Homo sapiens*. After each molecule was inserted into the Swiss Drug Design server, a sequence of possible targets to be used in our line of study was returned as a result. A dynamic table that classifies the possible targets predicted after each analysis for the query molecule, returning protein targets that are described by their name and the name of the gene.

The targets are classified on the basis of their classes, which the server summarizes in a pie chart that lists only the best classified targets. The server lists the 15 or 10 best similarity values according to the change and parameters of each search; in this work, we select the 10 best values. The web server returns the probability of proteins that are good targets in relation to the query molecule, which is assumed to be bioactive. For the analysis and selection of the best proteins to be used as promising targets for current research, we used the server’s interpretation standard, which consists of determining the known active compounds in each target that present the greatest similarity, with values above 0.65 for 2D visualizations or above 0.85 for 3D visualizations [108].

Cannabidiol activates cannabinoid receptors, although only cannabinoid receptor 2 alleviates RA, as supported by various studies [111]. The expression of the target arachidonate 5-lipoxygenase (5-LOX) is increased in RA patients, and the overexpression of 15-LOX during the progression of RA leads to the inhibition of chondrocyte proliferation and apoptosis [112]. Kara and colleagues reported that the mRNA expression of the NAD-dependent deacetylase sirtuin 2 (SIRT2) is reduced in patients with RA [113]. SIRT2 expression is associated with the expression of RA markers, and its dysregulation can aggravate the severity of arthritis in animal models [114]. N-arachidonyl glycine, which is an endocannabinoid (arachidonyl–amino acid conjugate), can alleviate inflammatory pain via a mechanism independent of cannabinoid receptors [115]. However, this endocannabinoid is not recommended for the treatment of RA [116]. DNA polymerase beta expression decreases in peripheral blood mononuclear cells (PBMCs) from patients with active RA and mice with collagen-induced arthritis (CIA). Additionally, DNA polymerase beta deficiency exacerbated LPS plus ATP-induced macrophage pyroptosis, whereas Pol β overexpression inhibited pyroptosis [117]. Therefore, the ability of cannabidiol to ameliorate RA may be associated with other CB2R-independent pathways.

Curcumin activates G-protein coupled receptor 97 (GPR97), which is bound to the Gi/Go subunit [118]. This receptor can regulate the migration of lymphatic endothelial cells [118]. Additionally, inhibitors of the curcumin target monoamine oxidase A have been proposed as pharmacological strategies for repurposing RA [119]. Prostaglandins (PGs) promote synovitis, cartilage degradation, bone resorption, and joint pain regulation. Therefore, PGs are potential therapeutic targets for treating RA [120]. Additionally, some data reveal a possible influence of prostaglandin E synthase (PTGES) polymorphisms on the pathogenesis of RA in terms of disease severity [121]. Thus, the possible inhibitory effect of curcumin on PTGES may be beneficial for RA treatment. Additionally, curcumin can activate LOX-5, causing negative effects on inflammation and RA [122].

In arthritis, some glycation products, such as FLs (Nε-(1-deoxyfructosyl)lysine) and AGEs (advanced glycation end products), are described. RAGE (receptor for AGEs) is a cell-surface receptor found in the synovial tissue of patients with RA, and its activation may decrease the expression of Glo1 (glyoxalase I) [123]. Curcumin inhibits Glo 1, and this effect can promote RA progression [124]. Compared with those in HCs, Nrf2 levels are elevated in patients with RA, and these proteins can be used as indicators to monitor disease activity and prognosis in patients with RA [125]. Nrf2 activates the antioxidant response, inhibiting the formation of free radicals/reactive oxygen species and inflammation. Thus, curcumin can activate Nrf2, reducing oxidative stress and hypothetically ameliorating rheumatoid arthritis [126,127]. Additionally, IκB kinase-1 and IκB kinase-2 regulate NF-κB activity and cytokine-induced NF-κB activation [128]. NF-κB is well recognized as a pivotal regulator of inflammation in RA. Therefore, curcumin-mediated inhibition of the NF-κB pathway can serve as a target in the treatment of RA [129,130]. In general, treatment with curcumin impairs RA development, although Glo-1 inhibition can induce a contrary pathway and should be narrowly studied.

Monoamine oxidase (MAO)-A and MAO-B are enzymes that may contribute to rheumatoid arthritis (RA) inflammation. MAO inhibitors may help relieve RA symptoms, such as pain and stiffness [131]. Curiously, curcumin inhibits MAO, which may explain part of the protective effects of curcumin in patients with RA [132].

Paeoniflorin can hypothetically act on galectin-3, and the activation of this adhesion molecule can induce cytokine and chemokine elevation in rheumatoid fibrocyte-like synoviocytes (FLSs) [133]. Moreover, the serum levels of galectin-3 were greater in RA patients than in controls and showed high diagnostic power for RA. Similarly, galectin-9 (Gal-9) expression is also increased in RA patients; thus, Gal-9 can be regarded as a new biomarker for evaluating RA activity and therapeutic effects after treatment with tacrolimus (TAC) [134]. Therefore, the possible inhibition of galectin-3 and 9 caused by paeoniflorin could ameliorate RA pathogenesis and represent a new therapeutic intervention.

Some studies have shown beneficial effects due to Hsp90 impairment [135]. RA patients exhibit pronounced anti-Hsp60, anti-Hsp70, and anti-Hsp90 humoral autoimmune responses independent of RA pathophysiology, although this humoral response may modulate the inflammatory status in this disease [136]. However, the relationships among Hsp90, paeoniflorin and other natural products in RA are unclear.

Norepinephrine (NE) release from synovial tyrosine hydroxylase-positive cells (TH+ cells) was lower in patients with RA than in those with OA. TH+ cells and the release of NE are related to massive synovial inflammation, in contrast to the results of in vitro assays indicating the anti-inflammatory properties of NE at relatively high concentrations (10^−5^ M). Therefore, NE secretion by TH+ cells may be an anti-inflammatory mechanism that counteracts local inflammation. Thus, TH+ cell-derived NE can be an important local factor of immunomodulation in synovial inflammation [137]. Additionally, norepinephrine has anti-inflammatory and chondroprotective effects on human osteoarthritic chondrocytes, reducing inflammatory RA pathology [138]. Paeoniflorin can induce the release of noradrenaline from noradrenergic nerve termini, and this mechanism may be related to norepinephrine transporter modulation [139].

The chronic synovial membrane (SM) of RA patients is rich in blood vessels [140], and other factors may increase SM vascularization [141]. Synovial angiogenesis is reduced in VEGF knockout mice in antigen-induced models of arthritis [142], which suggests that VEGF contributes to the pathology of RA. Additionally, VEGF and its receptors are detectable in serum and synovial fluids [143,144]. Thus, paeoniflorin can negatively regulate VEGF-A, attenuate angiogenesis and possibly alleviate RA. We found no clear relationship between paeoniflorin and somastatin receptors or the significant involvement of these receptors in AR.

Quercetin can reduce NADPH oxidase activity by decreasing superoxide anion (O2) generation [116]. NADPH oxidase 4 (NOX4) may contribute to the progression of RA, and NADPH oxidase subunits may be attractive therapeutic targets for RA [144]. Quercetin, a typical dietary flavonoid, is thought to exert antidepressant effects by inhibiting the monoamine oxidase-A (MAO-A) reaction [145]. Thus, MAO-A inhibition can favor RA development. The FMS-related tyrosine kinase 3 ligand (Flt3L)/CD135 axis promotes the proliferation and differentiation of dendritic cells, which play key roles in RA immunopathology. Treatment of RA patients with prednisolone and adalimumab inhibits the Flt3L/CD135 axis, and both substances represent novel therapeutic targets [146]. These findings indicate that quercetin may be tested with corticoids as an alternative therapeutic.

The increase in carbonic anhydrase (CA) II autoantibodies observed in RA patients increased erythrocyte oxidative stress and lipid peroxidation [147]. As quercetin can interact with carbonic anhydrase II (CA II) and inhibit its enzyme function [148], this may be a possible mechanism through which quercetin reverts RA.

The serine/threonine protein kinase PIM1 (PIM1) is upregulated in CD4+ T cells from patients with early RA. Treatment with small-molecule PIM inhibitors decreased the activation and proliferation of T-cell receptor-stimulated CD4+ T cells isolated from patients with early and untreated RA and reduced the production of proinflammatory cytokines. In vivo, treatment with PIM inhibitors reduced arthritis severity and cartilage destruction in mice with collagen-induced arthritis [149]. Quercetin is a potent inhibitor of PIM1 [150], and these interaction studies can be used to test other quercetin derivatives in RA patients. Another quercetin target is LOX-5, and some groups have described potent quercetin inhibition against this enzyme [151,152], which may be an effective pathway to reduce RA symptoms.

Dopaminergic agents have been used in the past for the treatment of RA because the stimulation of D2-like DR leads to the inhibition of prolactin, a proinflammatory hormone that is released by the anterior pituitary gland [153] and is present at high concentrations in the serum and synovial fluid of RA patients [154]. Therefore, the possible interaction between tetramethylpyrazine and dopamine receptors represents a new therapeutic strategy for treating RA.

Patients with rheumatoid arthritis present significant increases in the levels of alpha-1-antitrypsin, alpha-1-antichymotrypsin, and interalpha-trypsin inhibitors in the serum as well as in the synovial fluid of the subjects who were studied [155]. This approach revealed alpha 1 antitrypsin (A1AT) as a target of RA patients’ autoantibodies. Homocysteinylated A1AT was identified as a new possible antigenic target of autoantibodies in serum from RA patients, including RA-seronegative patients [156]. Additionally, trypsin and other proteolytic enzymes, such as bromelain and rutoside, which are orally administered, have been used for RA treatment [157]. Tetramethylpyrazine can interact with trypsin, as observed via spectroscopic analysis [158]. However, whether this interaction can promote a positive or negative result should be investigated. Chymotrypsin is a proteolytic enzyme that may help with rheumatoid arthritis (RA) by reducing inflammation [159].

Resveratrol competitively inhibits monoamine oxidase-A [160] and has antidepressant-like effects on the regulation of central serotonin and noradrenaline levels and the related MAO-A activities [161]. Carbonic anhydrase I (CA1) may contribute to bone loss in rheumatoid arthritis (RA) through the production of hydrogen ions. Some studies suggest that carbonic anhydrase inhibitors (CAIs) may help with RA by reducing bone resorption [162]. Additionally, in 2023, Khateri and collaborators reported the inhibitory effects of resveratrol on carbonic anhydrase activity [163]. Estrogen receptor alpha (ERα) is an intracellular receptor that may play a role in rheumatoid arthritis (RA) susceptibility and disease progression [164]. ERα expression in cartilage may contribute to the effects of estrogen on synovitis and joint destruction [165]. Dysregulation of estrogen receptor-related receptor-α (ERR-α) expression may contribute to bone and cartilage destruction in inflammatory arthritis. In one study, estradiol treatment decreased the frequency and severity of arthritis in a model of postmenopausal RA [166]. Therefore, as resveratrol acts on Erα to repress its function [167], this pathway may be a key element in the mechanism of resveratrol activity. Resveratrol has been shown to inhibit NF-κB activity through the above mechanism. Therefore, resveratrol can reduce the production of COX-2 and function as a powerful drug against RA [8,168,169].

Kaempferol inhibits NOX4 activity, and this effect reduces airway inflammation, increases autophagy [170], inhibits aldose reductase in models related to DR [171], inhibits tyrosinase activity and interacts with tyrosinase active binding sites [172]. Additionally, kaempferol inhibits carbonic anhydrase II [173], LOX-5, aryl hydrocarbon receptor (AhR) [174], and estradiol 17-beta-dehydrogenase 1 and 2 [175]; however, its relationship with RA has not been explored [176].

The other predicted targets include G-protein coupled receptor 55, glycine receptor subunit alpha-1, arachidonate 15-lipoxygenase; cathepsin D, thrombin, beta amyloid A4 protein, beta-secretase 1, cyclooxygenase 1, estradiol 17-betadehydrogenase 3, NADPH oxidase 4, vasopressin V2 receptor, aldose reductase, cytochrome P450 19A1; cytochrome P6, cytochrome P450 3A4; and cytochrome P450 2B6, potassium channel subfamily K member 2 (KCNK2), protein-tyrosine phosphatase 2C, heat shock factor protein 1, testis-specific androgen-binding protein, mineralocorticoid receptor, estradiol 17-beta-dehydrogenase 2, and estradiol 17-beta-dehydrogenase 1, which are not associated with RA pathogenesis or the studied isolated natural substances in the table.

Among the isolated compounds studied, tetramethylpyrazine is among the most promising candidates for the treatment of rheumatoid arthritis. The compound has a low molecular weight (136,198 Da) and moderate lipophilicity (LogP = 1.565), characteristics that may favor permeability through biological membranes and contribute to good bioavailability. Furthermore, it has a reduced number of hydrogen bond acceptors and an absence of rotatable bonds, indicating a relatively simple molecular structure, which may facilitate interactions with potential molecular targets.

From a pharmacokinetic point of view, tetramethylpyrazine showed good predicted permeability, a high free fraction in plasma, and no interaction with P-glycoprotein, which are factors that may favor its absorption and systemic availability. In terms of the toxicological profile, the compound did not predict cardiotoxicity associated with the hERG channel and demonstrated a low overall risk of ADMET, suggesting a relatively favorable safety profile.

Thus, considering all these predictive properties, tetramethylpyrazine has potential for further investigation in the context of rheumatoid arthritis treatment.

## 10. Conclusions

Herbal remedies can represent complementary alternatives in the treatment of rheumatoid arthritis (RA), mainly because of their ease of use, lower cost, and, in many cases, lower incidence of adverse effects than some conventional medications used to control the disease. Furthermore, natural sources, such as medicinal plants, have anti-inflammatory, antioxidant, antimicrobial, and healing properties, which can contribute to the modulation of the inflammatory response and the improvement of symptoms. The beneficial properties of natural products in relation to those of commercial medications are discussed in this review, highlighting that their use can aid in the treatment of RA. and potentially contributes to reducing the need for or dosage of drugs associated with greater side effects.

These natural products include copaiba oil, *Salix alba*, *Tripterygium wilfordii*, *Curcuma longa*, *Pterodon* spp., *Cannabis sativa*, *Chenopodium ambrosioides* L., and quercetin, in addition to some chemical constituents, such as sesquiterpenes, salicin, alkaloids, flavonoids and glycosides. Additionally, isolated substances, such as paeoniflorin, were also detected.

Although these products have proven anti-inflammatory effects, we observed differences between studies in terms of their toxic doses or side effects, such as fetal malformation. Furthermore, studies have reported drug interactions that induce nephrotoxicity and hemorrhage.

Although natural products have a low cost in RA treatment, their use should be further explored, especially in relation to dosages and drug combinations that may be harmful to patients.

Among the substances listed in this study for the treatment of RA, paeoniflorin, quercetin, resveratrol, and celastrol have already been evaluated in clinical trials and are considered adjunctive alternatives for the treatment of rheumatoid arthritis. These compounds have demonstrated therapeutic potential, especially because of their anti-inflammatory and immunomodulatory properties. However, studies are still ongoing, mainly because of the need for a better understanding of possible adverse effects, toxicity profiles, and pharmacokinetic and pharmacodynamic parameters, which are still poorly described in the literature.

In this review, we used in silico tools to predict the pharmacokinetic and toxicity data for natural compounds with potential for use in RA treatment to fill the gap in the knowledge of such parameters. The results indicated that curcumin, tetrametilpirazine, and resveratrol have the most promising pharmacokinetic and toxicological parameters, suggesting the high potential of these compounds as drugs.

## Figures and Tables

**Table 4:** The top ten predicted macromolecular targets for each compound were identified on the basis of their probability scores from the SwissTargetPrediction server, as shown in Table. Adapted from [108,109,110].

Isolated Substance	UniProt ID	Predicted Protein Target
Cannabidiol	P21554	Cannabinoid receptor 1
	P34972	Cannabinoid receptor 2
	Q9Y2T6	G-protein coupled receptor 55
	P09917	Arachidonate 5-lipoxygenase
	Q8IXJ6	NAD-dependent deacetylase sirtuin 2
	Q14330	N-arachidonyl glycine receptor
	P23415	Glycine receptor subunit alpha-1
	P06746	DNA polymerase beta (by homology)
	P16050	Arachidonate 15-lipoxygenase
	P07339	Cathepsin D
Curcumin	P21397	Monoamine oxidase A
	P05067	Beta amyloid A4 protein
	O14684	Prostaglandin E synthase
	P56817	Beta-secretase 1
	Q04760	Glyoxalase I
	Q16236	Nuclear factor erythroid 2-related factor 2
	P09917	Arachidonate 5-lipoxygenase
	P23219	Cyclooxygenase-1
	Q9Y6K9	Inhibitor of NF-kappa-B kinase (IKK)
	O14920	Estradiol 17-beta-dehydrogenase 3
Paeoniflorin	P17931	Galectin-3
	O00182	Galectin-9
	P07900	Heat shock protein HSP 90-alpha
	P23975	Norepinephrine transporter
	P35346	Somatostatin receptor 5
	P30874	Somatostatin receptor 2
	P31391	Somatostatin receptor 4
	P30872	Somatostatin receptor 1
	P32745	Somatostatin receptor 3
	P15692	Vascular endothelial growth factor A
Quercetin	Q9NPH5	NADPH oxidase 4
	P30518	Vasopressin V2 receptor
	P15121	Aldose reductase
	P21397	Monoamine oxidase A
	P36888	Tyrosine-protein kinase receptor FLT3
	P11511	Cytochrome P450 19A1
	P00734	Thrombin
	P00918	Carbonic anhydrase II
	P11309	Serine/threonine protein kinase PIM1
	P09917	Arachidonate 5-lipoxygenase
Tetramethylpyrazine	P14416	Dopamine D2 receptor (by homology)
	P15538	Cytochrome P450 11B1
	P19099	Cytochrome P450 11B2
	P11509	Cytochrome P450 2A6
	O95069	Potassium channel subfamily K member 2 (by homology)
	P00734	Thrombin
	P07477	Trypsin I
	Q99895	Chymotrypsin C
	P08684	Cytochrome P450 3A4
	P20813	Cytochrome P450 2B6
Resveratrol	P21397	Monoamine oxidase A
	P00918	Carbonic anhydrase II
	P03372	Estrogen receptor alpha
	P23219	Cyclooxygenase-1
	P23975	Norepinephrine transporter
	P35354	Cyclooxygenase-2
	P43166	Carbonic anhydrase VII
	P05067	Beta amyloid A4 protein
	P00915	Carbonic anhydrase I
	P07451	Carbonic anhydrase III
Celastrol	Q06124	Protein-tyrosine phosphatase 2C
	Q00613	Heat shock factor protein 1
	P15121	Aldose reductase
	P11511	Cytochrome P450 19A1
	P04278	Testis-specific androgen-binding protein
	Q2M385	Macrophage-expressed gene 1 protein
	P98170	Inhibitor of apoptosis protein 3
	Q92831	Histone acetyltransferase PCAF
	P30305	Dual specificity phosphatase Cdc25B
	P08235	Mineralocorticoid receptor
Kaempferol	Q9NPH5	NADPH oxidase 4
	P15121	Aldose reductase (by homology)
	P14679	Tyrosinase
	P36888	Tyrosine-protein kinase receptor FLT3
	P00918	Carbonic anhydrase II
	P09917	Arachidonate 5-lipoxygenase
	P43166	Carbonic anhydrase VII
	P37059	Estradiol 17-beta-dehydrogenase 2
	P14061	Estradiol 17-beta-dehydrogenase 1
	P35869	Aryl hydrocarbon receptor
Hesperidin	P11511	Cytochrome P450 19A1
	P18405	Steroid 5-alpha-reductase 1
	Q9NY91	Low affinity sodium-glucose cotransporter
	P13866	Sodium/glucose cotransporter 1
	P31639	Sodium/glucose cotransporter 2
	Q9HAS3	Solute carrier family 28 member 3
	O43570	Carbonic anhydrase XII
	P14679	Tyrosinase
	Q16678	Cytochrome P450 1B1
	P00918	Carbonic anhydrase II

## Data Availability

The data that support the findings of this study are available from the corresponding author upon reasonable request.

## Abbreviations

Alanine transaminase (ALT), Alanine phosphatase (AlkPhos), Alpha-linolenic acid (ALA), Arachidonic acid (AA), Aspartic acid transaminase (AST), Autoimmune rheumatic diseases (ARDs), Cannabidiol (CBD), C-Jun N-terminal kinase (JNK), Collagen-induced arthritis (CIA), Curcumin (Cur), Cyclooxygenase (COX), Disease-modifying antirheumatic drugs (DMARDs), Drug-induced liver injury (DILI), Erythrocyte sedimentation rate (ESR), Extracellular signal-regulated kinase (ERK), Feline immunodeficiency virus (FIV), Fibroblast-like synovial cells (FLS), Food and Drug Administration (FDA), Fraction unbound in human plasma (hum_Fup%), Blood-to-plasma concentration ratio in human (RBP), Gamma-glutamyltransferase (GGT), Glucocorticoids (GCs), Hesperidin (HES), Human Ether-a-go-Related Gene (hERG), Human jejunal effective permeability (cm/s × 10^4^) (Peff), I kappa B (IκB) kinase (IKK).

## References

[1] Martins M.A., Carrilho F.J., Alves V.A.F., Castilho E.A. (2016). Clínica Médica: Doenças Endócrinas e Metabólicas, Doenças Ósseas, Doenças Reumatológicas.

[2] Uke P., Maharaj A., Adebajo A. (2025). A review on the epidemiology of rheumatoid arthritis: An update and trends from current literature. Best Pract. Res. Clin. Rheumatol..

[3] American College of Rheumatology (2017). Rheumatoid Arthritis. https://www.rheumatology.org/I-Am-A/Patient-Caregiver/DiseasesConditions/Rheumatoid-Arthritis.

[4] Ventades N.G., Laza I.M., Hervella M., De-La-Rúa C. (2018). A recording form for differential diagnosis of arthropathies. Int. J. Paleopathol..

[5] Semerano L., Minichiello E., Bessis N., Boissier M.C. (2016). Novel Immunotherapeutic Avenues for Rheumatoid Arthritis. Trends Mol. Med..

[6] Kalden J.R. (2016). Emerging Therapies for Rheumatoid Arthritis. Rheumatol. Ther..

[7] Brahmachari G., Brahmachari G. (2019). Chapter 1—Discovery and development of anti-inflammatory agents from natural products: An overview. Natural Product Drug Discovery, Discovery and Development of Anti-Inflammatory Agents from Natural Products.

[8] Zhang Z., Liu Y., Liang X., Wang Q., Xu M., Yang X., Tang J., He X., He Y., Zhang D. (2025). Advances in nanodelivery systems based on apoptosis strategies for enhanced rheumatoid arthritis therapy. Acta Biomater..

[9] Dudics S., Langan D., Meka R.R., Venkatesha S.H., Berman B.M., Che C.T., Moudgil K.D. (2018). Natural Products for the Treatment of Autoimmune Arthritis: Their Mechanisms of Action, Targeted Delivery, and Interplay with the Host Microbiome. Int. J. Mol. Sci..

[10] De Salvo J.C., Skiba M.B., Howe C.L., Haiber K.E., Funk J.L. (2019). Natural Product Dietary Supplement Use by Individuals with Rheumatoid Arthritis: A Scoping Review. Arthritis Care Res..

[11] Dini V.S.Q., FurtaDo S.C., Barcellos J.F.M., Costa O.T.F. (2019). Ação anti-inflamatória do óleo de copaíba em artrite induzida em modelo animal: Uma Revisão Sistemática. Sci. Amazon. Ciências Biológicas..

[12] Coelho I.A.S., Carmo P.B., Santos N.S., Mariúba G.C.B., Rebelo M.A., Pereira M.D. (2018). O emprego de plantas medicinais nos casos de artrite reumatoide. Saúde Em Foco.

[13] Sung S., Kwon D., Um E., Kim B. (2019). Could Polyphenols Help in the Control of Rheumatoid Arthritis?. Molecules.

[14] Galati S., Di Stefano M., Martinelli E., Poli G., Tuccinardi T. (2021). Recent Advances in In Silico Target Fishing. Molecules.

[15] Paula D.C.C., Souza C.B.T. (2022). The Importance of The Rational Use of Medicinal Plants and Herbal Medicines in Brazil from the Perspective of Pharmaceutical Care. Rev. Ágora.

[16] Shin J.I., Jeon Y.J., Lee S., Lee Y.G., Kim J.B., Kwon H.C., Kim S.H., Kim I., Lee K., Han Y.S. (2018). Apoptotic and Anti-Inflammatory Effects of *Eupatorium Japonicum* Thunb. In Rheumatoid Arthritis Fibroblast-Like Synoviocytes. BioMed Res. Internat..

[17] Areosa V.B.M. (2019). Efeito do Óleo Essencial de Copaíba (*Copaifera multijuga* Hayne) Sobre a Morfologia Hepática e Cardíaca em um Modelo de Inflamação Induzida por Zymosan: Um Estudo Estereológico. 2019. 73 f. Dissertação (Mestrado em Imunologia Básica e Aplicada)—Universidade Federal do Amazonas, Manaus. https://tede.ufam.edu.br/handle/tede/7234.

[18] Lauron-Moreau A., Pitre F.E., Argus G.W., Labrecque M., Brouillet L. (2015). Phylogenetic relationships of american willows (*Salix* L., Salicaceae). PLoS ONE.

[19] Qadir A., Aqil M., Ali A., Ahmad F.J., Ahmad S., Arif M., Khan N. (2020). GC–MS analysis of the methanolic extracts of *Smilax China* and *Salix alba* and their antioxidant activity. Turk. J. Chem..

[20] Maistro E.L., Terrazzas P.M., Sawaya A.C.H.F., Rosa P.C.P., Perazzo F.F., de Mascarenhas I.O.G. (2022). In vivo toxicogenic potential of Salix alba (Salicaceae) bark extract. J. Toxicol. Environ. Health A.

[21] Lin C.-R., Tsai S.H.L., Wang C., Lee C.-L., Hung S.-W., Ting Y.-T., Hung Y.C. (2023). Willow Bark (*Salix* spp.) Used for Pain Relief in Arthritis: A Meta-Analysis of Randomized Controlled Trials. Life.

[22] Zhang Y., Mao X., Li W., Chen W., Wang X., Ma Z., Lin N. (2021). *Tripterygium wilfordii*: An inspiring resource for rheumatoid arthritis treatment. Med. Res. Rev..

[23] Chen S.R., Dai Y., Zhao J., Lin L., Wang Y., Wang Y. (2018). A mechanistic overview of triptolide and celastrol, natural products from *Tripterygium wilfordii* Hook F. Front. Pharmacol..

[24] Yang Y., Ye Y., Qiu Q., Xiao Y., Huang M., Shi M., Liang L., Yang X., Xu H. (2016). Triptolide inhibits the migration and invasion of rheumatoid fibroblast-like synoviocytes by blocking the activation of the JNK MAPK pathway. Int. Immunopharmacol..

[25] Mao N., Xie X. (2024). Mechanisms of Tripterygium wilfordii Hook F on treating rheumatoid arthritis explored by network pharmacology analysis and molecular docking. Open Med..

[26] Zhu Y., Zhang L., Zhang X., Wu D., Chen L., Hu C., Wen C., Zhou J. (2022). Tripterygium wilfordii glycosides ameliorates collagen-induced arthritis and aberrant lipid metabolism in rats. Front. Pharmacol..

[27] Zhou G.L., Su S.L., Yu L., Shang E.X., Hua Y.Q., Yu H., Duan J.A. (2025). Exploring the liver toxicity mechanism of Tripterygium wilfordii extract based on metabolomics, network pharmacological analysis and experimental validation. J. Ethnopharmacol..

[28] Hoscheid J., Cardoso M.L. (2015). Sucupira as a Potential Plant for Arthritis Treatment and Other Diseases. Arthritis.

[29] Pacheco M.T. Desenvolvimento e Caracterização de Nanoemulsão à Base de Óleo de Sucupira (*Pterodon* spp.), Avaliação de sua Toxicidade e Bioatividade in vitro. 2017. 96 f. Dissertação (Mestrado em Ciências Farmacêuticas)—Universidade Federal de Goiás, Goiânia, 2017. FLORIEN. Sucupira. https://florien.com.br/wp-content/uploads/2016/06/SUCUPIRA.pdf.

[30] Parisi V., Vassalo A., Pisano C., Signorino G., Cardile F., Sorrentino M., Colelli F., Fucci A., D’andrea E.L., De Tommasi N. (2020). A Herbal Mixture from Propolis, Pomegranate, and Grape Pomace Endowed with Anti-inflamatory Activity in In Vivo Rheumatoid Arthritis Model. Molecules.

[31] Heo Y.R., Son C.N., Baek W.K., Kim S.H. (2022). Grape seed proanthocyanidin extract induces apoptotic and autophagic cell death in rheumatoid arthritis fibroblast-like synoviocytes. Arch. Rheumatol..

[32] Nattagh-Eshtivani E., Pahlavani N., Ranjbar G., Gholizadeh Navashenaq J., Salehi-Sahlabadi A., Mahmudiono T., Nader Shalaby M., Jokar M., Nematy M., Barghchi H. (2022). Does propolis have any effect on rheumatoid arthritis? A review study. Food Sci. Nutr..

[33] Pereira W.S., Da Silva G.P., Vigliano M.V., Leal N.R.F., Pinto F.A., Fernandes D.C., Santos S.V.M., Martino T., Nascimento J.R., De Azevedo A.P.S. (2018). Anti-arthritic properties of crude extract from *Chenopodium ambrosioides* L. leaves. J. Pharm. Pharmacol..

[34] Dhande P.P., Gupta A., Jain S., Dawane J.S. (2017). Anti-inflammatory and Analgesic Activities of Tropical Formulations of *Pterocarpus santalinus* Powder in Rat Model of Chronic Inflammation. J. Clin. Diag. Res..

[35] Rosas E.C., Correa L.B., Pádua T.A., Costa T.E.M.M., Mazzei J.L., Heringer A.P., Bizarro C.A., Kaplan M.A., Figueiredo M.R., Henriques M.G. (2015). Anti-inflammatory Effect of *Schinus terebinthifolius* Raddi Hydroalcoholic Extract on Neutrophil Migration in Zymosan-Induced Arthritis. J. Ethnopharmacol..

[36] Chiang C.C., Li Y.R., Lai K.H., Cheng W.J., Lin S.C., Wang Y.H., Chen P.J., Yang S.H., Lin C.C., Hwang T.L. (2020). Aqueous Extract of Kan-Lu-Hsiao-Tu-Tan Ameliorates Collagen-Induced Arthritis in Mice by Inhibiting Oxidative Stress and Inflammatory Responses. Life.

[37] Elsohly M.A., Radwan M.M., Gul W., Chandra S., Galal A. (2017). Phytochemistry of *Cannabis sativa* L. Prog. Chem. Org. Nat. Prod..

[38] Good P., Haywood A., Gogna G., Martin J., Yates P., Greer R., Hardy J. (2019). Oral medicinal cannabinoids to relieve symptom burden in the palliative care of patients with advanced cancer: A double-blind, placebo controlled, randomized clinical trial of efficacy and safety of cannabidiol (CBD). BMC Palliat. Care.

[39] Bartner L.R., Mcgrath S., Rao S., Hyatt L.K., Wittenburg L.A. (2018). Pharmacokinetics of cannabidiol administered by 3 delivery methods at 2 different dosages to healthy dogs. Can. J. Vet. Res..

[40] Orrin D.A., Patel E., Thiele M., Wong R.A.C., Harden S., Greenwood G.M., Kenneth S. (2018). On behalf of the GWPCARE1 Part A Study Group. Randomized, dose-ranging safety trial of cannabidiol in Dravet syndrome. Neurology.

[41] Lowin T., Tingting R., Zurmahr J., Classen T., Schneider M., Pongratz G. (2020). Cannabidiol (CBD): A killer for inflammatory rheumatoid arthritis synovial fibroblasts. Cell Death Dis..

[42] Mujahid K., Rasheed M.S., Sabir A., Nam J., Ramzan T., Ashraf W., Imran I. (2025). Cannabidiol as an Immune Modulator: A Comprehensive Review. Saudi Pharm. J..

[43] Valentino R.J., Volkow N.D. (2024). Cannabis and Cannabinoid Signaling: Research Gaps and Opportunities. J. Pharmacol. Exp. Ther..

[44] Schouten M., Dalle S., Mantini D., Koppo K. (2024). Cannabidiol and brain function: Current knowledge and future perspectives. Front. Pharmacol..

[45] Silva D.M., Ferreira G.N., Papaccioli S.N., Coutinho Y.M.V., Dantas J.M., Gomes L.V., Vasconcelos F.G., Issy S.R., Gomes F.S., Martins J.L.R. (2025). Anti-Inflammatory and Immunomodulatory Potential of Cannabidiol in Rheumatoid Arthritis: Integrative Review. Front. J. Soc. Technol. Environ. Sci..

[46] Daily J.W., Yang M., Park S. (2016). Efficacy of Turmeric Extracts and Curcumin for Alleviating the Symptoms of Joint Arthritis: A Systematic Review and Meta-Analysis of Randomized Clinical Trials. J. Med. Food.

[47] Asteriou E., Gkoutzourelas A., Mavropoulos A., Katsiari C., Sakkas L.I., Bogdanos D.P. (2018). Curcumin for the Management of Periodontitis and Early ACPA-Positive Rheumatoid Arthritis: Killing Two Birds with One Stone. Nutrients.

[48] Liu A.J., Wu P.C., Chen Y.P., Chu H.-T., Chang H.-H. (2026). Effects of curcumin and *Curcuma longa* extract on inflammatory biomarkers in patients with rheumatoid arthritis (RA) and systemic lupus erythematosus (SLE): A systematic review and meta-analysis of randomized controlled trials. Inflamm. Res..

[49] Barbosa Retameiro A.C., Boaro C.T., Rodriguez D.F.S., Stein T., Andrade Menolli R., Ayala T.S., Panis C., Costa R.M., Bertolini G.R.F., Ribeiro L.d.F.C. (2025). Curcumin (*Curcuma longa*) Supplementation Improves Tibiofemoral Joint Outcomes in Low-Dose Prednisone-Treated Experimental Rheumatoid Arthritis. Microsc. Microanal..

[50] Laurindo L.F., de Carvalho G.M., de Oliveira Zanuso B., Figueira M.E., Direito R., de Alvares Goulart R., Buglio D.S., Barbalho S.M. (2023). Curcumin-Based Nanomedicines in the Treatment of Inflammatory and Immunomodulated Diseases: An Evidence-Based Comprehensive Review. Pharmaceutics.

[51] Xin Q., Yuan R., Shi W., Zhu Z., Wang Y., Cong W. (2019). A review for the anti-inflammatory effects of paeoniflorin in inflammatory disorders. Life Sci..

[52] Liu X., Wang Z., Qian H., Tao W., Zhang Y., Hu C., Mao W., Guo Q. (2022). Natural medicines of targeted rheumatoid arthritis and its action mechanism. Front. Immunol..

[53] Chen L., Qi H., Jiang D., Wang R., Chen A., Yan Z., Xiao J. (2013). The New Use of an Ancient Remedy: A Double-Blinded Randomized Study on the Treatment of Rheumatoid Arthritis. Am. J. Chin. Med..

[54] Jia Z., He J. (2016). Paeoniflorin ameliorates rheumatoid arthritis in rat models through oxidative stress, inflammation and cyclooxygenase 2. Exp. Ther. Med..

[55] Cheng C., Lin J.Z., Li L., Yang J.L., Jia W.W., Huang Y.H., Du F.F., Wang F.Q., Li M.J., Li Y.F. (2016). Pharmacokinetics and disposition of monoterpene glycosides derived from *Paeonia lactiflora* roots (Chishao) after intravenous dosing of antiseptic XueBiJing injection in human subjects and rats. Acta Pharmacol. Sin..

[56] Li X., Shi F., Zhang R., Sun C., Gong C., Jian L., Ding L. (2016). Pharmacokinetics, Safety, and Tolerability of Amygdalin and Paeoniflorin After Single and Multiple Intravenous Infusions of Huoxue-Tongluo Lyophilized Powder for Injection in Healthy Chinese Volunteers. Clin. Ther..

[57] Yang J., Wei Z., Li H., Lv S., Fu Y., Xiao L. (2024). Paeoniflorin inhibits the inflammation of rheumatoid arthritis fibroblast-like synoviocytes by downregulating hsa_circ_009012. Heliyon.

[58] Batiha G.E., Beshbishy A.M., Ikram M., Mulla Z.S., El-Hack M.E.A., Taha A.E., Algammal A.M., Elewa Y.H.A. (2020). The Pharmacological Activity, Biochemical Properties, and Pharmacokinetics of the Major Natural Polyphenolic Flavonoid: Quercetin. Foods.

[59] Moon Y.J., Wang L., Dicenzo R., Morris M.E. (2008). Quercetin pharmacokinetics in humans. Biopharm. Drug Dispos..

[60] Ferry D.R., Smith A., Malkhandi J., Fyfe D.W., Detakats P.G., Anderson D., Baker J., Kerr D.J. (1996). Phase I clinical trial of the flavonoid quercetin: Pharmacokinetics and evidence for in vivo tyrosine kinase inhibition. Clin. Cancer Res..

[61] Nguyen M.A., Staubach P., Wolffram S., Langguth P. (2015). The Influence of Single-Dose and Short-Term Administration of Quercetin on the Pharmacokinetics of Midazolam in Humans. Pharmacokinet. Pharmacodyn. Drug Transp. Metab..

[62] Zhang F., Zhang Y., Zhou J., Cai Y., Li Z., Sun J., Xie Z., Hao G. (2024). Metabolic effects of quercetin on inflammatory and autoimmune responses in rheumatoid arthritis are mediated through the inhibition of JAK1/STAT3/HIF-1α signaling. Mol. Med..

[63] Javadi F., Ahmadzadeh A., Eghtesadi S., Aryaeian N., Zabihiyeganeh M., Rahimi F.A., Jazayeri S. (2017). The Effect of Quercetin on Inflammatory Factors and Clinical Symptoms in Women with Rheumatoid Arthritis: A Double-Blind, Randomized Controlled Trial. J. Am. Coll. Nutr..

[64] Liu X., Tao T., Yao H., Zheng H., Wang F., Gao Y. (2023). Mechanism of action of quercetin in rheumatoid arthritis models: Meta-analysis and systematic review of animal studies. Inflammopharmacology.

[65] Zhu Y., Chen H., Yi Y., Zhou F., Zhou W., Zhong S. (2026). Recent developments in quercetin nanomedicine and applications in osteoarthritis and rheumatoid arthritis therapies. Front. Pharmacol..

[66] Chen L., Liu T., Wang Q., Liu J. (2017). Anti-inflammatory Effect of Combined Tetramethylpyrazine, Resveratrol and *Curcumin* in vivo. BMC Complement. Altern. Med..

[67] Amiot M.J., Romier B., Dao T.M., Fanciullino R., Ciccolini J., Burcelin R., Pechere L., Emond C., Savoure T.J.F., Seree E. (2013). Optimization of trans-Resveratrol bioavailability for human therapy. Biochimie.

[68] Azachi M., Yatuv R., Katz A., Hagay Y., Danon A. (2014). A novel red grape cells complex: Health effects and bioavailability of natural resveratrol. Int. J. Food Sci. Nutr..

[69] Banaszewska B., Wrotyńska-Barczyńska J., Spaczynski R.Z., Pawelczyk L., Duleba A.J. (2016). Effects of Resveratrol on Polycystic Ovary Syndrome: A Double-blind, Randomized, Placebo-controlled Trial. J. Clin. Endocrinol. Metab..

[70] Sattarinezhad A., Roozbeh J., Shirazi Yeganeh B., Omrani G.R., Shams M. (2019). Resveratrol reduces albuminuria in diabetic nephropathy: A randomized double-blind placebo-controlled clinical trial. Diabetes Metab..

[71] Aufschnaiter A., Kohler V., Khalifa S., Abd El-Wahed A., Du M., El-Seedi H., Büttner S. (2020). Apitoxin and Its Components against Cancer, Neurodegeneration and Rheumatoid Arthritis: Limitations and Possibilities. Toxins.

[72] Baek Y.H., Huh J.E., Lee J.D., Choi D.Y., Park D.S. (2006). Antinociceptive effect and the mechanism of bee venom acupuncture (Apipuncture) on inflammatory pain in the rat model of collagen-induced arthritis: Mediation by alpha2-Adrenoceptors. Brain Res..

[73] Park H.J., Son D.J., Lee C.W., Choi M.S., Lee U.S., Song H.S., Lee J.M., Hong J.T. (2007). Melittin inhibits inflammatory target gene expression and mediator generation via interaction with IkappaB kinase. Biochem. Pharmacol..

[74] Hartmann A.D., Wilhelm N., Erfle V., Hartmann K. (2016). Clinical efficacy of melittin in the treatment of cats infected with the feline immunodeficiency virus. Tieraerztliche Prax. Ausg. Kleintiere Heimtiere.

[75] Huang R., He X., Meng Q., Yan G., Dong K., Tian Y. (2025). Melittin: A promising therapeutic agent for rheumatoid arthritis treatment. Toxicon.

[76] Pareek A., Mehlawat K., Tripathi K., Pareek A., Chaudhary S., Ratan Y., Apostolopoulos V., Chuturgoon A. (2024). Melittin as a therapeutic agent for rheumatoid arthritis: Mechanistic insights, advanced delivery systems, and future perspectives. Front. Immunol..

[77] Venkatesha S.H., Dudics S., Astry B., Moudgil K.D. (2016). Control of autoimmune inflammation by celastrol, a natural triterpenoid. Pathog. Dis..

[78] Xiao S., Zhang M., Liang Y., Wang D. (2017). Celastrol synergizes with oral nifedipine to attenuate hypertension in preeclampsia: A randomized, placebo-controlled, and double blinded trial. J. Am. Soc. Hypertens..

[79] Devi K.P., Malar D.S., Nabavi S.F., Sureda A., Xiao J., Nabavi S.M., Daglia M. (2015). Kaempferol and inflammation: From chemistry to medicine. Pharmacol. Res..

[80] Yoon H.-Y., Lee E.-G., Lee H., Cho I.J., Choi Y.J., Sung M.-S., Yoo H.-G., Yoo W.-H. (2013). Kaempferol inhibits IL-1_-induced proliferation of rheumatoid arthritis synovial fibroblasts and the production of cox-2, pge2 and mmps. Int. J. Mol. Med..

[81] Wang H., Xia G., Guan X., Wang L., Qin L., Fu M. (2022). Expression of Nrf2 protein in serum of patients with rheumatoid arthritis: A novel indicator for disease activity and disease prognosis. Clin. Biochem..

[82] Burak C., Wolffram S., Zur B., Langguth P., Fimmers R., Alteheld B., Stehle P., Egert S. (2019). Effect of alpha-linolenic acid in combination with the flavonol quercetin on markers of cardiovascular disease risk in healthy, nonobese adults: A randomized, double-blinded placebo-controlled crossover trial. Nutrition.

[83] Nazir M.M., Farzeen I., Saeed S., Ashraf A. (2025). Kaempferol as a multitargeted phytotherapeutic for arthritis: Systematic review and meta-analysis of preclinical models. Inflammopharmacology.

[84] Hong H.M., Zhou J.W., Li M.Y., Hao G.F., Xie Z.J. (2025). Kaempferol-A Natural Drug for Rheumatoid Arthritis. Chin. J. Integr. Med..

[85] Cao F., Cheng M.H., Hu L.Q., Shen H.H., Tao J.H., Li X.M., Pan H.F., Gao J. (2020). Natural products action on pathogenic cues in autoimmunity: Efficacy in systemic lupus erythematosus and rheumatoid arthritis as compared to classical treatments. Pharmacol. Res..

[86] Qi W., Lin C., Fan K., Chen Z., Liu L., Feng X., Zhang H., Shao Y., Fang H., Zhao C. (2019). Hesperidin inhibits synovial cell inflammation and macrophage polarization through suppression of the PI3K/AKT pathway in complete Freund’s adjuvant-induced arthritis in mice. Chem. Biol. Interact..

[87] Cheraghpour M., Imani H., Ommi S., Alavian S.M., Karimi-Shahrbabak E., Hedayati M., Yari Z., Hekmatdoost A. (2019). Hesperidin improves hepatic steatosis, hepatic enzymes, and metabolic and inflammatory parameters in patients with nonalcoholic fatty liver disease: A randomized, placebo-controlled, double-blind clinical trial. Phytother. Res..

[88] Qin Z., Chen L., Liu M., Tan H., Zheng L. (2019). Hesperidin reduces adverse symptomatic intracerebral hemorrhage by promoting TGF-β1 for treating ischemic stroke using tissue plasminogen activator. Neurol. Sci..

[89] Kometani T., Fukuda T., Kakuma T., Kawaguchi K., Tamura W., Kumazawa Y., Nagata K. (2008). Effects of alpha-glucosylhesperidin, a bioactive food material, on collagen-induced arthritis in mice and rheumatoid arthritis in humans. Immunopharmacol. Immunotoxicol..

[90] Corsale I., Carrieri P., Martellucci J., Piccolomini A., Verre L., Rigutini M., Panicucci S. (2018). Flavonoid mixture (diosmin, troxerutin, rutin, hesperidin, quercetin) in the treatment of I-III degree hemorroidal disease: A double-blind multicenter prospective comparative study. Int. J. Color. Dis..

[91] Tabeshpour J., Hosseinzadeh H., Hashemzaei M., Karimi G. (2020). A review of the hepatoprotective effects of hesperidin, a flavanon glycoside in citrus fruits, against natural and chemical toxicities. DARU J. Pharm. Sci..

[92] Long Z., Xiang W., He Q., Xiao W., Wei H., Li H., Guo H., Chen Y., Yuan M., Yuan X. (2023). Efficacy and safety of dietary polyphenols in rheumatoid arthritis: A systematic review and meta-analysis of 47 randomized controlled trials. Front. Immunol..

[93] Marangon C.A., Martins V.C., Leite P.M., Santos D.A., Nitschke M., Plepis A.M.G. (2017). Chitosan/gelatin/copaiba oil emulsion formulation and its potential on controlling the growth of pathogenic bacteria. Ind. Crops Prod..

[94] Pascoal D.R.C., Velozo E.S., Braga M.E.M., Sousa H.C., Cabral-Albuquerque E.C.M., De Melo S.A.B.V. (2020). Bioactive compounds of *Copaifera* sp. impregnated into three-dimensional gelatin dressings. Drug Deliv. Transl. Res..

[95] Símaro G.V., Lemos M., Silva J.J.M.D., Cunha W.R., Carneiro L.J., Ambrósio S.R., Cunha N.L., De Andrade S.F., Arruda C., Banderó-Filho V.C. (2022). In vivo study of anti-inflammatory and antinociceptive activities of *Copaifera* pubiflora Benth oleoresin. Nat. Prod. Res..

[96] Ames-Sibin A.P., Barizão C.L., Castro-Ghizoni C.V., Silva F.M.S., Sá-Nakanishi A.B., Bracht L., Bersani-Amado C.A., Marçal-Natali M.R., Bracht A., Comar J.F. (2018). β-Caryophyllene, the major constituent of copaiba oil, reduces systemic inflammation and oxidative stress in arthritic rats Ana, P.J. Cell. Biochem..

[97] Fidyt K., Fiedorowicz A., Strządała L., Szumny A. (2016). β-caryophyllene and β-caryophyllene oxide-natural compounds of anticancer and analgesic properties. Cancer Med..

[98] Ames-Sibin A.P., Chagas-Almeida A.C., Souza A.B.P., Andrade A.P.M., Castro J.C., Ferreira S.B.S., Silva-Comar F.M.S., Cuman R.K.N., Bruschi M.L., Natali M.R.M. (2025). Copaiba essential oil carried in a self-nanoemulsifying drug delivery system improves adjuvant-induced arthritis in rats. J. Pharm. Pharmacol..

[99] Ministry of Health (BR) (2015). Monograph of the Species Salix alba (White willow).

[100] Marchi J.P., Tedesco L., Melo ADa C., Frasson A.C., França V.F., Sato S.W., Lovato E.C.W. (2016). *Curcuma longa* L., o açafrão da terra, e seus benefícios medicinais. Arq. Cienc. Saúde UNIPAR Umuarama.

[101] Vieira A.R.M., Teixeira L.H.S., Santos W.P. (2017). *Cannabis sativa*: O Consumo de Canabidiol no Tratamento da Epilepsia. Revista Saúde em Foco—Edição n° 10—Ano: 2018 revistaonline@unifia.edu.br Página 903. http://jornal.faculdadecienciasdavida.com.br/index.php/RBCV/article/download/500/139/.

[102] Vallianatou T., Giaginis C., Tsantili-Kakoulidou A. (2015). The impact of physicochemical and molecular properties in drug design: Navigation in the “drug-like” chemical space. Adv. Exp. Med. Biol..

[103] Garcia-Cortes M., Robles-Diaz M., Stephens C., Ortega-Alonso A., Lucena M.I., Andrade R.J. (2020). Drug induced liver injury: An update. Arch. Toxicol..

[104] Chalasani N., Bonkovsky H.L., Fontana R., Lee W., Stolz A., Talwalkar J., Reddy K.R., Watkins P.B., Navarro V., Barnhart H. (2015). Features and Outcomes of 899 Patients with Drug-Induced Liver Injury: The DILIN Prospective Study. Gastroenterology.

[105] Boelsterli U.A. (2003). Diclofenac-induced liver injury: A paradigm of idiosyncratic drug toxicity. Toxicol. Appl. Pharmacol..

[106] Tu C., Gao Y., Song D., Niu M., Ma R.R., Zhou M.X., He X., Xiao X.H., Wang J.B. (2021). Screening for Susceptibility-Related Biomarkers of Diclofenac-Induced Liver Injury in Rats Using Metabolomics. Front. Pharmacol..

[107] Krause F., Voigt K., Di Ventura B., Öztürk M.A. (2023). Reverse Dock: A web server for blind docking of a single ligand to multiple protein targets using Auto Dock Vina. Front. Mol. Biosci..

[108] Daina A., Michielin O., Zoete V. (2019). Swiss Target Prediction: Updated data and new features for efficient prediction of protein targets of small molecules. Nucl. Acids Res..

[109] Daina A., Zoete V. (2024). Testing the predictive power of reverse screening to infer drug targets, with the help of machine learning. Comms. Chem..

[110] Gfeller D., Michielin O., Zoete V. (2013). Shaping the interaction landscape of bioactive molecules. Bioinformatics.

[111] Gui H., Tong Q., Qu W., Mao C., Dai S. (2015). The endocannabinoid system and its therapeutic implications in rheumatoid arthritis. Int. Immunopharmacol..

[112] Bryda J., Watroba S. (2018). The proinflammatory role of lipoxygenases in rheumatoid arthritis. J. Pre-Clin. Clin. Res..

[113] Kara M., Yolbaş S., Şahin C., Koca S.S. (2017). Changes in sirtuin 2 and sirtuin 3 mRNA expressions in rheumatoid arthritis. Eur. J. Rheumatol..

[114] Zhao T., Alam H.B., Liu B., Bronson R.T., Nikolian V.C., Wu E., Chong W., Li Y. (2015). Selective Inhibition of SIRT2 Improves Outcomes in a Lethal Septic Model. Curr. Mol. Med..

[115] Succar R., Mitchell V.A., Vaughan C.W. (2007). Actions of N-arachidonyl-glycine in a rat inflammatory pain model. Mol. Pain.

[116] Burstein S.H. (2018). *N*-Acyl Amino Acids (Elmiric Acids): Endogenous Signaling Molecules with Therapeutic Potential. Mol. Pharmacol..

[117] Gu L., Sun Y., Wu T., Chen G., Tang X., Zhao L., He L., Hu Z., Sun L., Pan F. (2022). A novel mechanism for macrophage pyroptosis in rheumatoid arthritis induced by Pol β deficiency. Cell Death Dis..

[118] Valtcheva N., Primorac A., Jurisic G., Hollmén M., Detmar M. (2013). The orphan adhesion G protein-coupled receptor GPR97 regulates migration of lymphatic endothelial cells via the small GTPases RhoA and Cdc42. J. Biol. Chem..

[119] Sur D., Dutta A., Mondal C., Banerjee A., Haldar P.K., Maji H.S., Bala A. (2022). Repurposing monoamine oxidase inhibitors (MAOI) for the treatment of rheumatoid arthritis possibly through modulating reactive oxidative stress mediated inflammatory cytokines. Inflammopharmacology.

[120] Ma Y., Hong F.-F., Yang S.-L. (2021). Role of prostaglandins in rheumatoid arthritis. Clin. Exp. Rheumatol..

[121] Korotkova M., A Daha N., Seddighzadeh M., Ding B., I Catrina A., Lindblad S., Huizinga T.W.J., Toes R.E.M., Alfredsson L., Klareskog L. (2011). Variants of gene for microsomal prostaglandin E2 synthase show association with disease and severe inflammation in rheumatoid arthritis. Eur. J. Hum. Genet..

[122] Hong J., Bose M., Ju J., Ryu J.-H., Chen X., Sang S., Lee M.-J., Yang C.S. (2004). Modulation of arachidonic acid metabolism by curcumin and related beta-diketone derivatives: Effects on cytosolic phospholipase A(2), cyclooxygenases and 5-lipoxygenase. Carcinogenesis.

[123] Ahmed U., Thornalley P.J., Rabbani N. (2014). Possible role of methylglyoxal and glyoxalase in arthritis. Biochem. Soc. Trans..

[124] Liu M., Yuan M., Luo M., Bu X., Luo H.-B., Hu X. (2010). Binding of curcumin with glyoxalase I: Molecular docking, molecular dynamics simulations, and kinetics analysis. Biophys. Chem..

[125] Wang L., Lee I.M., Zhang S.M., Blumberg J.B., Buring J.E., Sesso H.D. (2009). Dietary intake of selected flavonols, flavones, and flavonoid-rich foods and risk of cancer in middle-aged and older women. Am. J. Clin. Nutr..

[126] Chadha S., Behl T., Kumar A., Khullar G., Arora S. (2020). Role of Nrf2 in rheumatoid arthritis. Curr. Res. Transl. Med..

[127] Ashrafizadeh M., Ahmadi Z., Mohammadinejad R., Farkhondeh T., Samarghandian S. (2020). Curcumin Activates the Nrf2 Pathway and Induces Cellular Protection Against Oxidative Injury. Curr. Mol. Med..

[128] Aupperle K.R., Bennett B.L., Han Z., Boyle D.L., Manning A.M., Firestein G.S. (2001). NF-κB Regulation by IκB Kinase-2 in Rheumatoid Arthritis Synoviocytes. J. Immunol..

[129] Esmaealzadeh N., Miri M.S., Mavaddat H., Peyrovinasab A., Zargar S.G., Kabiri S.S., Razavi S.M., Abdolghaffari A.H. (2024). The regulating effect of curcumin on NF-κB pathway in neurodegenerative diseases: A review of the underlying mechanisms. Inflammopharmacology.

[130] Xia Z.-B., Meng F.-R., Fang Y.-X., Wu X., Zhang C.-W., Liu Y., Liu D., Li G.-Q., Feng F.-B., Qiu H.-Y. (2018). Inhibition of NF-κB signaling pathway induces apoptosis and suppresses proliferation and angiogenesis of human fibroblast-like synovial cells in rheumatoid arthritis. Medicine.

[131] Won W., Choi H.-J., Yoo J.-Y., Kim D., Kim T.Y., Ju Y., Park K.D., Lee H., Jung S.Y., Lee C.J. (2022). Inhibiting peripheral and central MAO-B ameliorates joint inflammation and cognitive impairment in rheumatoid arthritis. Exp. Mol. Med..

[132] Juvekar A.R., Khatri D.K. (2016). Kinetics of Inhibition of Monoamine Oxidase Using Curcumin and Ellagic Acid. Pharmacogn. Mag..

[133] Filer A., Bik M., Parsonage G.N., Fitton J., Trebilcock E., Howlett K., Cook M., Raza K., Simmons D.L., Thomas A.M.C. (2009). Galectin 3 induces a distinctive pattern of cytokine and chemokine production in rheumatoid synovial fibroblasts via selective signaling pathways. Arthritis Rheum..

[134] Sun J., Sui Y., Wang Y., Song L., Li D., Li G., Liu J., Shu Q. (2021). Galectin-9 expression correlates with therapeutic effect in rheumatoid arthritis. Sci. Rep..

[135] Fouani M., Basset C.A., Mangano G.D., Leone L.G., Lawand N.B., Leone A., Barone R. (2022). Heat Shock Proteins Alterations in Rheumatoid Arthritis. Int. J. Mol. Sci..

[136] Mantej J., Polasik K., Piotrowska E., Tukaj S. (2019). Autoantibodies to heat shock proteins 60, 70, and 90 in patients with rheumatoid arthritis. Cell Stress Chaperones.

[137] Miller L.E., Grifka J., Schölmerich J., Straub R.H. (2002). Norepinephrine from synovial tyrosine hydroxylase positive cells is a strong indicator of synovial inflammation in rheumatoid arthritis. J. Rheumatol..

[138] Lorenz J., Schäfer N., Bauer R., Jenei-Lanzl Z., Springorum R., Grässel S. (2016). Norepinephrine modulates osteoarthritic chondrocyte metabolism and inflammatory responses. Osteoarthr. Cartil..

[139] Liu T.P., Liu I.M., Tsai C.C., Lai T.Y., Hsu F.L., Cheng J.T. (2002). Stimulatory effect of paeoniflorin on the release of noradrenaline from ileal synaptosomes of guinea-pig in vitro. J. Pharm. Pharmacol..

[140] Storgard C.M., Stupack D.G., Jonczyk A., Goodman S.L., Fox R.I., Cheresh D.A. (1999). Decreased angiogenesis and arthritic disease in rabbits treated with an alphavbeta3 antagonist. J. Clin. Investig..

[141] Koch A.E. (1998). Review: Angiogenesis: Implications for rheumatoid arthritis. Arthritis Rheum..

[142] Mold A.W., Tonks I.D., Cahill M.M., Pettit A.R., Thomas R., Hayward N.K., Kay G.F. (2003). Vegfb gene knockout mice display reduced pathology and synovial angiogenesis in both antigen-induced and collagen-induced models of arthritis. Arthritis Rheum..

[143] Lee S.S., Joo Y.S., Kim W.U., Min D.J., Min J.K., Park S.H., Cho C.S., Kim H.Y. (2001). Vascular endothelial growth factor levels in the serum and synovial fluid of patients with rheumatoid arthritis. Clin. Exp. Rheumatol..

[144] Sánchez M., Galisteo M., Vera R., Villar I.C., Zarzuelo A., Tamargo J., Pérez-Vizcaíno F., Duarte J. (2006). Quercetin downregulates NADPH oxidase, increases eNOS activity and prevents endothelial dysfunction in spontaneously hypertensive rats. J. Hypertens..

[145] Lee H.R., Yoo S.J., Park C.G., Kim J., Yoo I.S., Kang S.W. (2019). NADPH Oxidase 4 Regulates the Migration and Invasion of Synoviocytes in Rheumatoid Arthritis Through Pro-angiogenic Factor Secretion. Arthrits & Rheumatology.

[146] Bandaruk Y., Mukai R., Kawamura T., Nemoto H., Terao J. (2012). Evaluation of the inhibitory effects of quercetin-related flavonoids and tea catechins on the monoamine oxidase-A reaction in mouse brain mitochondria. J. Agric. Food Chem..

[147] Ramos M.I., Perez S.G., Aarrass S., Helder B., Broekstra P., Gerlag D.M., Reedquist K.A., Tak P.P., Lebre M.C. (2013). FMS-related tyrosine kinase 3 ligand (Flt3L)/CD135 axis in rheumatoid arthritis. Arthritis Res. Ther..

[148] Alver A., Şentürk A., Çakirbay H., Menteşe A., Gökmen F., Keha E.E., Uçar F. (2011). Carbonic anhydrase II autoantibody and oxidative stress in rheumatoid arthritis. Clin. Biochem..

[149] Khateri S., Mehrabi M., Khodarahmi R. (2023). Inhibitory effects of quercetin and resveratrol and their sulfonamide derivatives on the carbonic anhydrase activity: Spectroscopic studies of the binding process. Chem. Pap..

[150] Onuora S. (2021). Repurposed drugs could target PIM kinases in early RA. Nat. Rev. Rheumatol..

[151] Rathi A., Chaudhury A., Anjum F., Ahmad S., Haider S., Khan Z.F., Taiyab A., Chakrabarty A., Islam A., Hassan M.I. (2024). Targeting prostate cancer via therapeutic targeting of PIM-1 kinase by Naringenin and Quercetin. Int. J. Biol. Macromol..

[152] Kwon O.S., Choi J.S., Islam M.N., Kim Y.S., Kim H.P. (2011). Inhibition of 5-lipoxygenase and skin inflammation by the aerial parts of Artemisia capillaris and its constituents. Arch. Pharm. Res..

[153] Borbulevych O.Y., Jankun J., Selman S.H., Skrzypczak-Jankun E. (2004). Lipoxygenase interactions with natural flavonoid, quercetin, reveal a complex with protocatechuic acid in its X-ray structure at 2.1 A resolution. Proteins.

[154] McMurray R.W. (2001). Bromocriptine in rheumatic and autoimmune diseases. Semin. Arthritis Rheum..

[155] Fojtíková M., Tomasová Studýnková J., Filková M., Lacinová Z., Gatterová J., Pavelka K., Vencovský J., Senolt L. (2010). Elevated prolactin levels in patients with rheumatoid arthritis: Association with disease activity and structural damage. Clin. Exp. Rheumatol..

[156] Brackertz D., Hagmann J., Kueppers F. (1975). Proteinase inhibitors in rheumatoid arthritis. Ann. Rheum. Dis..

[157] Colasanti T., Atinelli D.S., Mancone C., Pecani A., Speziali M., Vomero M., Barbati C., Celia A., Finucci A., Perricone C. (2019). Homocysteinylated Alpha 1 Anti-Trypsin as a Potential Antigenic Target in Rheumatoid Arthritis. Ann. Rheum. Dis..

[158] Kulkarni A.P., Bendrey H.A. (2021). Systemic enzyme therapy with trypsin, bromelain and rutoside in the management of arthritis: An overview. Int. J. Res. Orthop..

[159] Shuai L., Chen Z., Fei P., Wang Q., Yang T. (2014). Spectroscopic analysis on the interaction of ferulic acid and tetramethylpyrazine with trypsin. Luminescence.

[160] Li J., Wang L., Zeng G., Li H., Luo J., Tian Q., Zhang Z. (2022). Chymotrypsin attenuates adjuvant-induced arthritis by downregulating TLR4, NF-κB, MMP-1, TNF-α, IL-1β, and IL-6 expression in Sprague—Dawley rats. Immunopharmacol. Immunotoxicol..

[161] Han Y.N., Ryu S.Y., Han B.H. (1990). Antioxidant Activity Resveratrol Closely Correlates with Its Monoamine oxidase-A Inhibitory Activity. Arch. Pharmacal Res..

[162] Yu Y., Wang R., Chen C., Du X., Ruan L., Sun J., Li J., Zhang L., O’Donnell J.M., Pan J. (2013). Antidepressant-like effect of trans-resveratrol in chronic stress model: Behavioral and neurochemical evidence. J. Psychiatr. Res..

[163] Chang X., Zheng Y., Yang Q., Wang L., Pan J., Xia Y., Yan X., Han J. (2012). Carbonic anhydrase I (CA1) is involved in the process of bone formation and is susceptible to ankylosing spondylitis. Arthritis Res. Ther..

[164] Engdahl C., Börjesson A.E., Forsman H.F., Andersson A., Stubelius A., Krust A., Chambon P., Islander U., Ohlsson C., Carlsten H. (2014). The role of total and cartilage-specific estrogen receptor alpha expression for the ameliorating effect of estrogen treatment on arthritis. Arthritis Res. Ther..

[165] Bonnelye E., Laurin N., Jurdic P., Hart D.A., Aubin J.E. (2008). Estrogen receptor-related receptor-α (ERR-α) is dysregulated in inflammatory arthritis. Rheumatology.

[166] Engdahl C., Jochems C., Windahl S.H., Börjesson A.E., Ohlsson C., Carlsten H., Lagerquist M.K. (2010). Amelioration of collagen-induced arthritis and immune-associated bone loss through signaling via estrogen receptor alpha, and not estrogen receptor beta or G protein-coupled receptor 30. Arthritis Rheum..

[167] Nwachukwu J.C., Srinivasan S., Bruno N.E., Parent A.A., Hughes T.S., Pollock J.A., Gjyshi O., Cavett V., Nowak J., Garcia-Ordonez R.D. (2014). Resveratrol modulates the inflammatory response via an estrogen receptor-signal integration network. Elife.

[168] Tsai M.H., Hsu L.F., Lee C.W., Chiang Y.C., Lee M.H., How J.M., Wu C.M., Huang C.L., Lee I.T. (2017). Resveratrol inhibits urban particulate matter-induced COX-2/PGE2 release in human fibroblast-like synoviocytes via the inhibition of activation of NADPH oxidase/ROS/NF-κB. Int. J. Biochem. Cell Biol..

[169] Yang C.M., Chen Y.W., Chi P.L., Lin C.C., Hsiao L.D. (2017). Resveratrol inhibits BK-induced COX-2 transcription by suppressing acetylation of AP-1 and NF-κB in human rheumatoid arthritis synovial fibroblasts. Biochem. Pharmacol..

[170] Xu J., Yu Z., Li W. (2023). Kaempferol inhibits airway inflammation induced by allergic asthma through NOX4-Mediated autophagy. Hum. Exp. Toxicol..

[171] Julius A., Renuka R.R., Hopper W., Babu Raghu P., Rajendran S., Srinivasan S., Dharmalingam K., Alanazi A.M., Arokiyaraj S., Prasath S. (2022). Inhibition of Aldose Reductase by Novel Phytocompounds: A Heuristic Approach to Treating Diabetic Retinopathy. Evid. Based Complement Altern. Med..

[172] Tsai C.-H., Liou Y.-L., Li S.-M., Liao H.-R., Chen J.-J. (2025). Antioxidant, Anti-α-Glucosidase, Anti-Tyrosinase, and Anti-Acetylcholinesterase Components from Stem of *Rhamnus formosana* with Molecular Docking Study. Antioxidants.

[173] Şentürk M., Gülçin İ., Beydemir Ş., Küfrevioğlu Ö.İ., Supuran C.T. (2011). In Vitro Inhibition of Human Carbonic Anhydrase I and II Isozymes with Natural Phenolic Compounds. Chem. Biol. Drug Des..

[174] Hui W., Dai Y. (2020). Therapeutic potential of aryl hydrocarbon receptor ligands derived from natural products in rheumatoid arthritis. Basic Clin. Pharmacol. Toxicol..

[175] Guo Q., Zheng K., Fan D., Zhao Y., Li L., Bian Y., Qiu X., Liu X., Zhang G., Ma C. (2017). Wu-Tou Decoction in Rheumatoid Arthritis: Integrating Network Pharmacology and In Vivo Pharmacological Evaluation. Front. Pharmacol..

[176] Sroka Z., Sowa A., Dryś A. (2017). Inhibition of lipoxygenase and peroxidase reaction by some flavonols and flavones: The structure-activity relationship. Nat. Prod. Commun..

